# Molecular Insights into the pH-Dependent Adsorption and Removal of Ionizable Antibiotic Oxytetracycline by Adsorbent Cyclodextrin Polymers

**DOI:** 10.1371/journal.pone.0086228

**Published:** 2014-01-21

**Authors:** Yu Zhang, Xiyun Cai, Weina Xiong, Hao Jiang, Haitong Zhao, Xianhai Yang, Chao Li, Zhiqiang Fu, Jingwen Chen

**Affiliations:** Key Laboratory of Industrial Ecology and Environmental Engineering (Ministry of Education), School of Environmental Science and Technology, Dalian University of Technology, Dalian, China; Queen's University at Kingston, Canada

## Abstract

Effects of pH on adsorption and removal efficiency of ionizable organic compounds (IOCs) by environmental adsorbents are an area of debate, because of its dual mediation towards adsorbents and adsorbate. Here, we probe the pH-dependent adsorption of ionizable antibiotic oxytetracycline (comprising OTCH_2_^+^, OTCH^±^, OTC^−^, and OTC^2−^) onto cyclodextrin polymers (CDPs) with the nature of molecular recognition and pH inertness. OTCH^±^ commonly has high adsorption affinity, OTC^−^ exhibits moderate affinity, and the other two species have negligible affinity. These species are evidenced to selectively interact with structural units (e.g., CD cavity, pore channel, and network) of the polymers and thus immobilized onto the adsorbents to different extents. The differences in adsorption affinity and mechanisms of the species account for the pH-dependent adsorption of OTC. The mathematical equations are derived from the multiple linear regression (MLR) analysis of quantitatively relating adsorption affinity of OTC at varying pH to adsorbent properties. A combination of the MLR analysis for OTC and molecular recognition of adsorption of the species illustrates the nature of the pH-dependent adsorption of OTC. Based on this finding, γ-HP-CDP is chosen to adsorb and remove OTC at pH 5.0 and 7.0, showing high removal efficiency and strong resistance to the interference of coexisting components.

## Introduction

**Figure 1:**
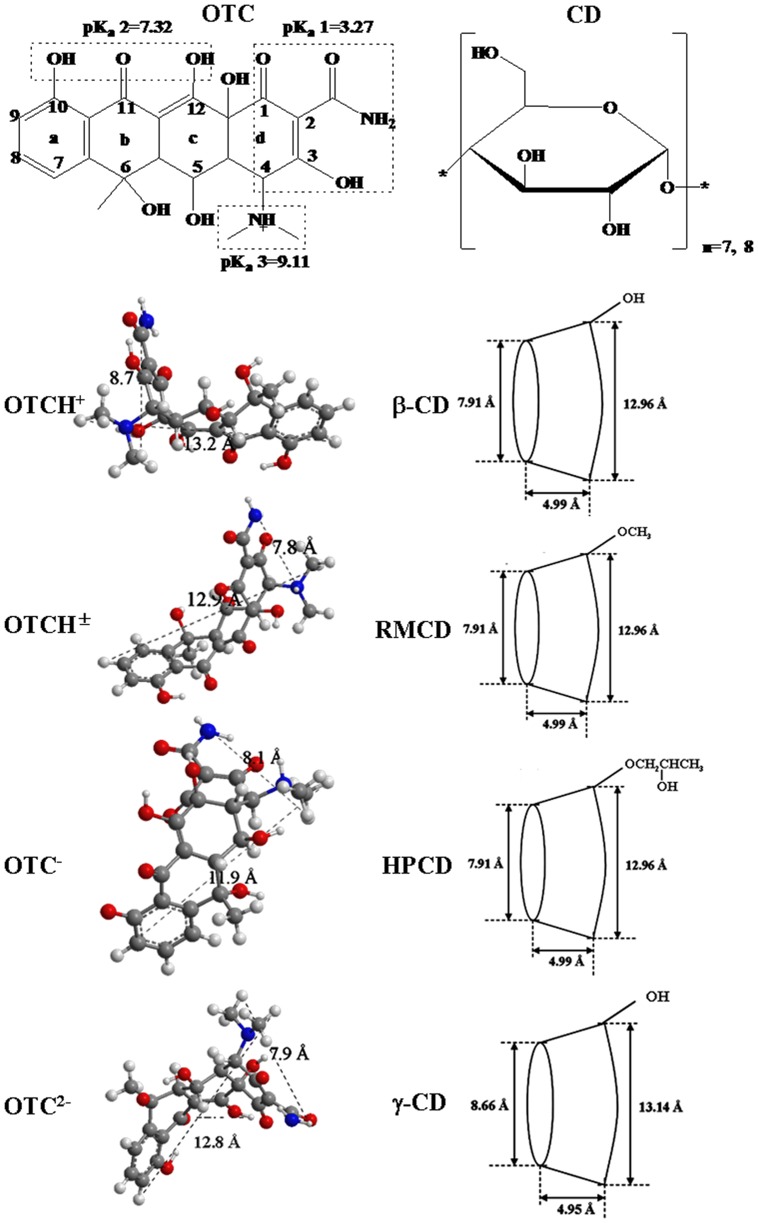
Molecular geometry of OTC species and CD [39].

Ionizable organic compounds (IOCs) occupy a large fraction of the pre-registered REACH compounds [1]. They have one or more p*K*_a_ values and are present in the form of a mixture of ionized and unionized species. The coexistence of these species is pH-dependent, which complicates the removal of IOCs in aquatic systems [1], [2]. Particularly, veterinary antibiotics (e.g., oxytetracycline (Figure 1)), one emerging IOCs, are ubiquitously detected [3] and have aroused serious concerns about the spread of antibiotic resistance genes [4], [5].

Adsorption is a widely used approach in the industry to remove organic pollutants from water [6], [7]. This technique is effective for antibiotic removal [7]. Commonly, adsorption efficiency of antibiotics is controlled by solution pH. It is probably due to dual mediation of pH toward antibiotics and adsorbents. Specifically, the solution pH mediates the fraction of the species by altering ionization degree of antibiotics. The species of many antibiotics (e.g., tetracyclines [8], [9], sulfathiazole [10] and sulfamethazine [11]) have been proven to differ in adsorption affinity. Meanwhile, conventional natural and synthetic adsorbents themselves (e.g., clays [8], [12], humic substances [13], [14], activated carbons [15], and CNTs [16]) probably suffer changes in structure and/or chemistry from the variation of pH. In turn, these changes may render alteration of adsorption sites of adsorbents. The dual mediation of pH complicates adsorption behavior of antibiotics, hereby making an impediment to the understanding of their pH-dependent adsorption.

Cyclodextrin polymers (CDPs) are growingly concerned environmental adsorbents, because they are endowed with a unique property of molecular recognition [17], [18]. They are widely applied in adsorption and removal of various organic pollutants [19]–[26]. The polymers are composed of functional unit (i.e., CD monomer) and cross-linking agents (e.g., epichlorohydrin (EPI)). CD monomer with an axial open cavity of hydrophobic character (Figure 1) is capable of including organics (or a moiety) in terms of geometric compatibility [27]–[29]. This inclusion is driven by various intermolecular interactions (e.g., hydrogen bonding, hydrophobic interactions, van der Waals forces, and electrostatic interactions) [30]–[32], indicating a unique property of molecular recognition. For example, the species of some IOCs with one or two p*K*_a_ values are included by CD in different patterns [33], [34]. This uniqueness is proved to remain in the polymers and considered as the primary adsorption mechanism [17]–[19].

Furthermore, the cross-linking agent EPI containing two reactive functional groups can form bonds with CD monomer and/or itself. The as-prepared polymer is a mixture of different CD units joined by repeating glyceryl linkers, representing a three-dimensional polymer network [18], [19], [21]. As the linkages of CD moieties are relatively polar, the polymer is highly hydrophilic and readily swells in water. This character makes adsorption sites of the polymer (e.g., CD cavity, pore channel, and network) easily accessible [18], [19], [21]. Additionally, the polymers are stable in both acidic and alkaline solutions [21], showing poor susceptibility to pH variations. It is due to less susceptibility of CD (p*K*_a_ = ca. 12) and isopropyl alcohol transformed by EPI (p*K*_a_ = ca. 17.1) to ionization under general conditions.

With these advantages, CDPs are suitable as model adsorbent for illustrating adsorption behavior of IOCs at varying pH. Therefore, this study was aimed to probe the pH-dependent adsorption of oxytetracycline (OTC, as model compound) onto CDPs and thus to select CDPs for removing the antibiotic. To this end, we investigated inclusion complexation (i.e., binding constants and complexation ratios) of CD and OTC through a spectroscopic titration technique and address CD inclusion mechanisms for the species on the basis of structural characterization and molecular simulation of the complexes. We conducted batch adsorption experiments of OTC onto CDPs for measuring adsorption affinity of OTC over pH. Mathematical equations for relating adsorption affinity of OTC at varying pH (or the species) to adsorbent properties were derived to reveal the nature of pH-dependent adsorption. The equations were used to select CDPs to remove OTC at different pH from water.

## Materials and Methods

### Materials

Oxytetracycline dihydrate (OTC, purity >95%) was obtained from AF Pharma LLC (US). Information on four cyclodextrin monomers (i.e., β-CD, RMCD, HPCD and γ-CD) were listed in Table S1. β-Cyclodextrin was recrystallized twice in deionized water and dried under vacuum. Other cyclodextrins were used as received. Other chemicals used were of analytical reagent grade.

Adsorbent CDPs were synthesized in our previous work [19] where the ratio of cyclodextrin to cross-linking agent EPI was set at 1∶5. They comprised four onefold polymers (i.e., β-CDP, γ-CDP, HP-CDP and RM-CDP) with single CD as complex and three composite polymers (i.e., β-γ-CDP, β-HP-CDP and γ-HP-CDP) with an equimolar mixture of two CDs as double complexes. Their physiochemical properties (including CD content, cross-linking degree, swelling ratio, particle size, pore size, surface area, and pore volume) were characterized and compiled in Table 1 in reference 19 [19]. The parameter CD content of the as-prepared polymers varied from 38.62% to 71.91%, depending on type and number of the complex used. The corresponding cross-linking degree, representing the mean amounts of EPI per CD unit, was in the range of 19.82 to 4.57.

**Table 1:** 1∶1 inclusion constants (*K*_C_, L/mol) of CD with the species^a^.

	β-CD	RMCD	HPCD	γ-CD
OTCH_2_^+^	712	2768	-b	-
OTCH^±^	554	54	259	128
OTC^−^	-	408	891	-
OTC^2−^	200	-	-	1098

^a^ The *K*_C_ of the species was obtained on the basis of the spectroscopic measurement of apparent *K*_C_ values of OTC and LINGO optimization;

^b^ approximately zero.

### Inclusion complexation between CD and OTC at varying pH

The solutions of OTC and CD were adjusted to five pH values ranging from 4.17 to 9.30, by adding appropriate amounts of 1-mol/L HCl or NaOH. The concentration of OTC was 20 µmol/L. A series of concentrations of CD were set, e.g., 0 to 2000 µmol/L for β-CD, 0 to 5200 µmol/L for RMCD and HPCD, and 0 to 6000 µmol/L for γ-CD. The solutions were incubated at room temperature (ca. 15°C) and protected from light. Absorption spectra of the solutions were recorded by a UV-2300 spectrophotometer (Techcomp Limited, Shanghai, China). Peak wavelengths varied from 353 nm to 372 nm, depending on pH. Data were used to obtain inclusion constant (*K*_C_, L/mol) and inclusion ratio *n* (namely a ratio of CD to OTC) in terms of the extended Benesi-Hildebrand equation (Equation 1) [35]. The parameter *n* is commonly 1/2, 1 or 2, which should yield the highest values of fitting degree of the equation. As *n* is determined, the parameter *K*_C_ can be calculated as a ratio of the intercept to the slope of the corresponding equation (Equation 1).
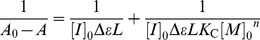
(1)where *A*_0_ and *A* are the absorbance of OTC and the mixture at peak wavelength, respectively; [*I*]_0_ and [*M*]_0_ are the initial concentrations of OTC and CD, respectively; 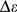 is the difference of the molar absorptivities of complexed and free OTC; *L* is optical distance; and *n* is inclusion ratio.

Moreover, inclusion complexes of CD with OTC were prepared by the grinding method [36]. Briefly, equivalent molar mixes of OTC and CD were grinded for 1 h with addition of three-fold deionized water. The complexes were lyophilized and characterized by FTIR (Shimadzu IRprestige-21, Japan) and NMR (Varian INOVA 400 MHz, USA). The complexes were prepared as KBr disks for FTIR measurement. All NMR spectra of the complexes were recorded in D_2_O with the chemical shift of HOD as reference.

### Adsorption of OTC onto cyclodextrin polymers at varying pH

Batch adsorption experiments were conducted in 25-mL glass centrifuge tubes and at 25°C, with a solid-liquid ratio of 1∶50. Concentrations of OTC varied from 1 to 30 mg/L. The solutions were adjusted to five pH values (varying from 4.65 to 10.11) by adding 1-mol/L HCl or NaOH. Appropriate amounts of 3-mol/L NaCl were added to all the solutions to obtain 0.02 mol/L Na^+^ or Cl^−^, in order to minimize interference from changes of ionic strength upon the pH adjustment. Then the solutions were kept in the dark and shaken by a rotary shaker (180 rpm) for 30 min. The 30-min contact time was sufficient to ensure adsorption equilibrium on the basis of the adsorption kinetic experiments (Text S1). The samples were centrifuged at 4000 rpm for 15 min. A drop of 6-mol/L HCl was added to the supernatant (acidified to pH ca. 2.5), in order to prevent OTC decomposition (Text S2). The supernatant was filtered through a 0.45-µm Millipore membrane. OTC in the filter was measured on an L-2000 HPLC (Hitachi, Japan). The stationary phase was a Hypersil ODS C-18 column (4.6×250 mm, 5 µm). The mobile phase comprised 22% acetonitrile and 78% NaH_2_PO_4_-H_3_PO_4_ buffer solution (pH 2.30). The flow rate was set at 1.0 mL/min. The column oven was maintained at 25°C. The injection volume was 10 µL. The detection wavelength was set at 355 nm. OTC was eluted at about 5 min. Data was processed with Langmuir and Freundlich models to obtain adsorption constants (Text S1). And the adsorption distribution coefficients (*K*_d_, L/Kg) were also calculated (Text S1).

Additional adsorption experiments were carried out at three pH values (i.e., 5.0, 7.0 and 9.0) for all the polymers, to confirm loading of OTC at varying pH onto the adsorbents. The initial concentration of OTC was set as 20 mg/L. Once approaching adsorption equilibrium, the polymers were centrifuged and rinsed with 10-mL deionized water. Then, the polymers were lyophilized for FTIR characterization.

### Computational simulation of inclusion complexation of CD and OTC

The atomic coordinates of β-CD (refcode POBRON) and γ-CD (refcode CIWMIE10) were selected from Cambridge Structural Database (CSD). Starting geometries of RMCD and HPCD molecules were built on the basis of the structure of β-CD using Chem3D Ultra software (version 8.0.3). RMCD was an optional derivative of β-CD with methylation of all OH groups at C2, C3 and C6 positions (Figure 1). HPCD was the hydropropylated derivative at C2 position (Figure 1) with substitution degree of 6.4 (Table S1). Starting geometries of the species of OTC were also built using the same software. Energy minimization of all the molecules was separately performed using PM3 semi-empirical method of Gaussian 09 software [37]. Based on the optimized geometries, molecular docking between CD and the species of OTC was carried out on CDOCKER module of Discovery Studio (DS, version 2.5) with the CHARMm force field. Ten most stable poses are obtained by the molecular docking technology. Among them, the pose of CD-OTC in which inclusion sites and binding orientation are most consistent with those of the complex evidenced by FTIR and NMR characterization would be chosen for further optimization. The selected complex structures were put into Solvation module of DS with the CHARMM force field to simulate the real water environment. The obtained structure then was optimized using Minimization and Minimization (QM-MM) protocols of DS. For the resulting structures, intermolecular interactions involved in the inclusion were discriminated using the Multiwfn 2.4 package developed by Lu and Chen [38]. In addition, energies of van der Waals forces and electrostatic interactions were recorded from the docking results of DS with the consideration of solvent contributions.

### Computational simulation of interactions of EPI oligomers with OTC

The polymer actually is a mixture of the materials comprising various ratios of CD to EPI, since the polymerization is rapid and relatively directionless. Due to the complexity of the cross-linking reaction, the structure of cyclodextrin polymers remains ambiguous. Here, we constructed some oligomers of EPI (e.g., ring-opened, tricyclo-, pentacyclo- and heptacyclo-ones) that are essential units of network and pore channel of the polymers. These oligomers may be indicative of structural units of the polymers. And hence, they were used as structure mode to bind the species of OTC. Their geometries were optimized with the aforesaid method for revealing molecular interactions between the polymer and guest compound in a qualitative way.

### Data analysis

All experiments were performed in triplicate and the resulting means were used in the following model analysis (Equation 2). Notably, the stability of OTC was ensured under all experimental circumstances (Text S2). The antibiotic comprises four species (i.e., OTCH_2_^+^, OTCH^±^, OTC^−^ and OTC^2−^) with three p*K*_a_ (i.e., 3.27, 7.32 and 9.11). Apparent interaction constants of OTC (i.e., CD inclusion and adsorption affinity) are considered as overall contribution of the four species (Equation 2). In Equation 2, the fractions of the species can be calculated by substituting pH and p*K*_a_ into Equations S1–S4 (Text S3). To reduce system error and randomness in the pH designation, we used LINGO 9.0 optimization software (Lingo System Inc., USA) to obtain the interaction constants of the species. The model LINGO1 was chosen and all coefficients were constrained to be ≥0 (Text S3). The interaction constants were non-weighted. The application of the LINGO software would be tested by comparing the apparent interaction constants calculated on the basis of Equation 2 and obtained from the respective experiments.
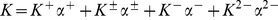
(2)where *K* is the apparent interaction constant of OTC; *K* ^+^, *K* ^±^, *K* ^−^ and *K* ^2−^ are the interaction constants of OTCH_2_^+^, OTCH^±^, OTC^−^ and OTC^2−^, respectively; and α^+^, α^±^, α^−^ and α^2−^ are corresponding mass fractions of the species.

## Results and Discussion

### Inclusion of CD with the species of OTC

**Figure 2:**
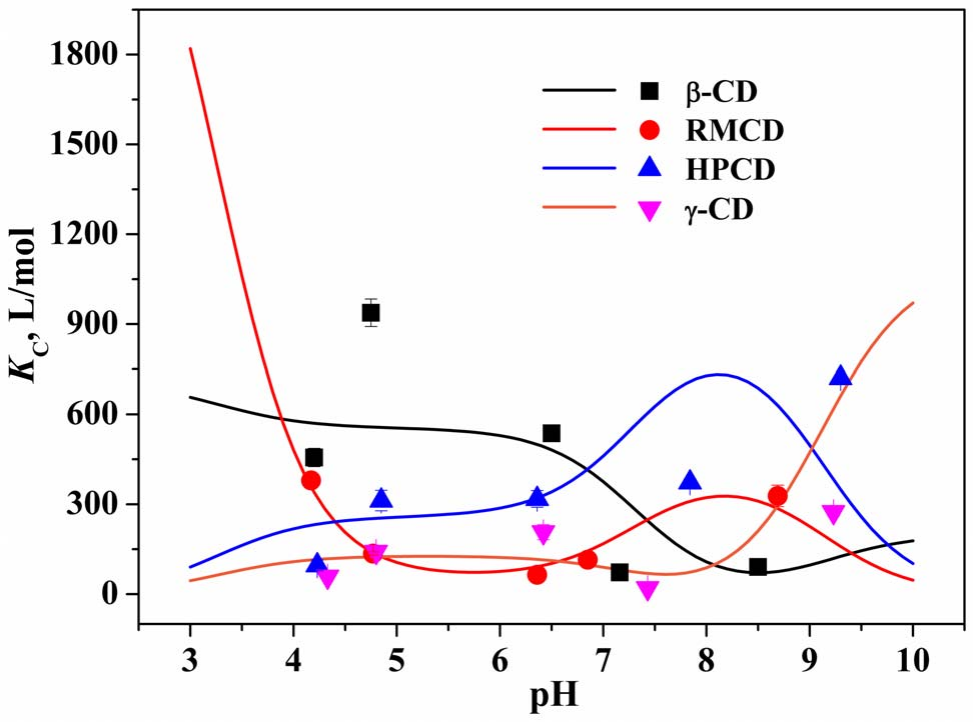
pH-dependent inclusion constants of OTC. The scatter represents the experimental values at certain pH and the line represents the calculated ones at varying pH on the basis of Equation 2.

Addition of CD caused obvious changes in the UV-vis spectrum of OTC (Figure S1), evidencing the formation of inclusion complex. Comparison of regression coefficients (*R* ^2^) derived from Equation 1 showed the complex had an inclusion ratio of 1∶1 (Table S2). The corresponding inclusion constant (i.e., *K*_C_) was compiled in Figure 2 and Table S2, relating to both CD type and solution pH. For instance, the inclusion capacity of β-CD with OTC, initially increased with pH increasing, approached the maximum at pH 4.75, and subsequently reduced. An inverse profile of inclusion capacity over pH, however, was observed for RMCD with the minimum at pH 6.36.

The *K*_C_ value of the species (Table 1) was obtained through the LINGO optimization of apparent *K*_C_ values for each CD at varying pH. A good linear relationship (*R* ^2^≥0.74 and P<0.05) was observed between the apparent *K*_C_ derived from spectroscopic titration experiments and calculated on the basis of Equation 2 into which the *K*_C_ and fraction of the species were substituted (Table S3). This relationship evidenced that the apparent *K*_C_ of OTC at varying pH (lines in Figure 2) could be obtained by Equation 2. Comparison of the *K*_C_ values (Table 1) demonstrated that molecular recognition of the species was related to cavity size [39] (e.g., β-CD versus γ-CD) and structural modification (β-CD versus HPCD/RMCD) of CD (Figure 1). It is due to the fact that the species differing in geometry (Figure 1) theoretically match the cavity of CD in different patterns. Overall, the differences in the *K*_C_ values of the species account for the pH-dependent inclusion of OTC, taking into account the nature of the pH inertness of CD.

### Inclusion mechanisms of CD with the species of OTC

Both FTIR and NMR characterization also convinced inclusion complexation of CD (e.g., β-CD) with the antibiotic. In the FTIR spectrum of the complex (Figure S2), stretching vibration peaks of O-H (3488 cm^−1^) and dimethylamino groups (3372 cm^−1^) of OTC turned to a strong and sharp peak at 3370 cm^−1^. In the NMR spectrum of the complex (Table S4), chemical shift of the protons at C4 and C6 of the OTC molecule transported from *δ*2.686 and *δ*1.601 to *δ*2.698 and *δ*1.635, respectively. And the concomitants were both downshield shift of H1–H6 protons of β-CD and reduction of splitting numbers of H3 peak (Table S4, Figure S3). The deshielding effect of β-CD is commonly attributable to the formation of hydrogen bonding. These two results clearly reveal that dimethylamino and its adjacent hydroxyl (i.e., C3, C5, C6 and/or C12) of the molecule are involved in the inclusion by forming hydrogen bonding.

The inclusion complex was visualized using molecular docking technique (Figure S4). At least four interactions (i.e., hydrogen bonding, hydrophobic interactions, van der Waals forces, and electrostatic interactions) are identified to form inclusion complexes but to different extents. Specifically, hydrogen bonding was observed to form between hydroxyl group/ether bond at the wide rim of β-CD and C5/C12-hydroxyl groups of all the species with an exception of OTC^−^. It supported the deshielding effect of CD in the NMR characterization. The number of hydrogen bonding involved followed an order of OTCH_2_^+^ (2)>OTCH^±^ (1) = OTC^2−^ (1)>OTC^−^ (0)) (Figure S4), which was in accordance with the decreasing order of *K*_C_ values (i.e., OTCH_2_^+^>OTCH^±^>OTC^2−^>OTC^−^ (Table 1)). It can be concluded that hydrogen bonding plays the predominant role in the inclusion complexation of CD with the antibiotic. It was worthy noting that OTCH^±^ with less solubility and higher hydrophobicity [40] had stronger inclusion potential than OTC^2−^, though both species had the same number of hydrogen bonding. It was ascribed to the presence of hydrophobic interactions that commonly are recognized as driving forces for the inclusion [31]. Furthermore, total interaction energy of the complex, denoted a sum of van der Waals forces and electrostatic interactions, followed an order of OTC^2−^>OTC^−^>OTCH_2_^+^>OTCH^±^ that is independent of the *K*_C_ of the species (Table S5, Figure S5). This finding implied that neither van der Waals forces nor electrostatic interactions played significant roles in the inclusion.

Few attempts to investigate CD inclusion of IOCs with one- or two-p*K*_a_ also demonstrate that the molecular recognition of the species involves various intermolecular interactions depending on CD type and compound structure [41]–[44]. Sebestyen and coworkers summarize that the differences in the *K*_C_ of the species for 8 amino acids are a consequence of the coordination of hydrogen bonding, steric effects and electrostatic interactions [42]. For 3 aromatic amino acids, phenylalanine, tyrosine and tryptophan, the *K*_C_ is in the range of 80–120 L/mol for the anion while it is much smaller for the zwitterion and negligible for the protonated species. However, the differences were less pronounced for the other five amino acids.

### Adsorption of OTC onto CDPs

The kinetics of adsorption was fast and the plateau reached within 15–30 min (Figure S6). The data of adsorption kinetics was well fitted to pseudo-second order model (Figure S7) relative to pseudo-first order model (Table S6). The rate controlling step was recognized as intraparticle diffusion for RMCDP and γ-HP-CDP on the basis of the moving boundary model (Text S1) while liquid film diffusion for the other five polymers (Table S7). It is mainly due to rather high solubility [40] and thus hydrophilic character of OTC, limiting mass transfer of the antibiotic to hydrophobic surfaces of materials.

**Figure 3:**
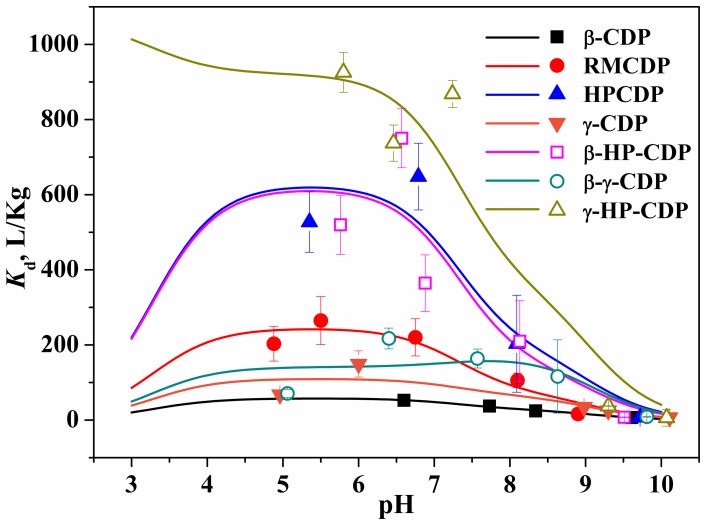
pH-dependent adsorption affinity of OTC onto CDPs. The scatter represents the experimental values at certain pH and the line represents the calculated ones at varying pH on the basis of Equation 2.

Figure S8 represented adsorption isotherms of OTC. The adsorption constants derived from Langmuir (*K*_L_) and Freundlich (*K*_F_) models were presented in Tables S8 and S9, respectively. In some cases, the sign of *K*_L_ is negative (Table S8), indicating the poor application of Langmuir model. In contrast, Freundlich model provided good fit to all data with *R* ^2^≥0.92 (Table S9). The values of heterogeneity factor (*n*) of Freundlich model ranged from 0.59 to 2.04 (Table S9). Obviously, most of them deviated from unit (*n* = 1) at which the Freundlich isotherm translates to a linear relationship identical to the linear isotherm governed by hydrophobic partitioning. Such deviation demonstrated that the OTC adsorption process was governed by complicated interactions other than hydrophobic interactions. Even for the same adsorbent, the values of *n* also varied with solution pH, indicating that adsorption interactions should differ at varying pH. The resulting *K*_F_ was of dimension, so that it would be a poor indicator for adsorption potential (Table S9). Instead of *K*_F_, the dimensionless *K*_d_, defined as the mean of *K*_d_ at all OTC levels (Text S1), was used to represent adsorption affinity of OTC in this study. Comparison of the *K*_d_ values indicated that this term varied with pH and the maxima commonly approached at pH 5.5–7.5 (Figure 3). Here, the *K*_d_ values were comparable to those reported for OTC onto other adsorbents, such as montmorillonite, kaolinite and goethite (ca. 769, 100 and 900 L/Kg at pH 6.5–6.7) [45], and native montmorillonite (ca. 1000 L/Kg at pH 5.0) [46].

### Selective adsorption and immobilization of the species

The term *K*_d_ of the species (Table 2) was obtained through the LINGO optimization of the apparent *K*_d_ values of OTC at varying pH (Figure 3). There was good similarity between the apparent *K*_d_ of OTC calculated on the basis of Equation 2 and obtained from batch adsorption experiments (Table S10). OTCH^±^ commonly had higher *K*_d_ values than OTC^−^, with an exception of adsorbent β-γ-CDP. By contrast, the *K*_d_ values of OTCH_2_^+^ and OTC^2−^ approached zero, except OTCH_2_^+^ onto γ-HP-CDP with a *K*_d_ value of 1063 L/Kg.

**Table 2:** Adsorption affinity (*K*_d_, L/Kg) of the species onto CDPs.

	β-CDP	RMCDP	HPCDP	γ-CDP	β-HP-CDP^b^	β-γ-CDP^b^	γ-HP-CDP^b^
OTCH_2_^+^	-a	-	-	-	-	-	1063
OTCH^±^	58	246	629	111	620	141	922
OTC^−^	27	67	178	63	137	170	342
OTC^2−^	-	-	-	-	-	-	-

^a^ approximately zero.

^b^ The composite polymer was synthesized with an equimolar mixture of two CDs as double complexes.

FTIR characterization of the adsorbents (e.g., β-CDP) with OTC treatments at three pH values (i.e., pH 5.0, 7.0 and 9.0) further demonstrated selective adsorption and thus immobilization of the species onto the polymers (Figure S9). Specifically, stretching vibration band of hydroxyl of the polymer transferred from 3435 cm^−1^ to 3385, 3415 and 3448 cm^−1^ with OTC treatments at pH 5.0, 7.0 and 9.0, respectively. Stretching band of C-OH at 1034 cm^−1^ was widened for OTC treatments at pH 5.0 and 7.0, while it disappeared for that at pH 9.0. Moreover, C-O-C stretching vibration shifted from 1154 cm^−1^ to 1158 cm^−1^ (pH 5.0), 1160 cm^−1^ (pH 7.0) and 1165 cm^−1^ (pH 9.0), respectively. The species-specific adsorption may be attributed to both ionization of OTC at varying pH and different adsorption interactions of the species, similar to the finding of the pH-dependent interactions of OTC in clay and organic matter [46].

**Figure 4:**
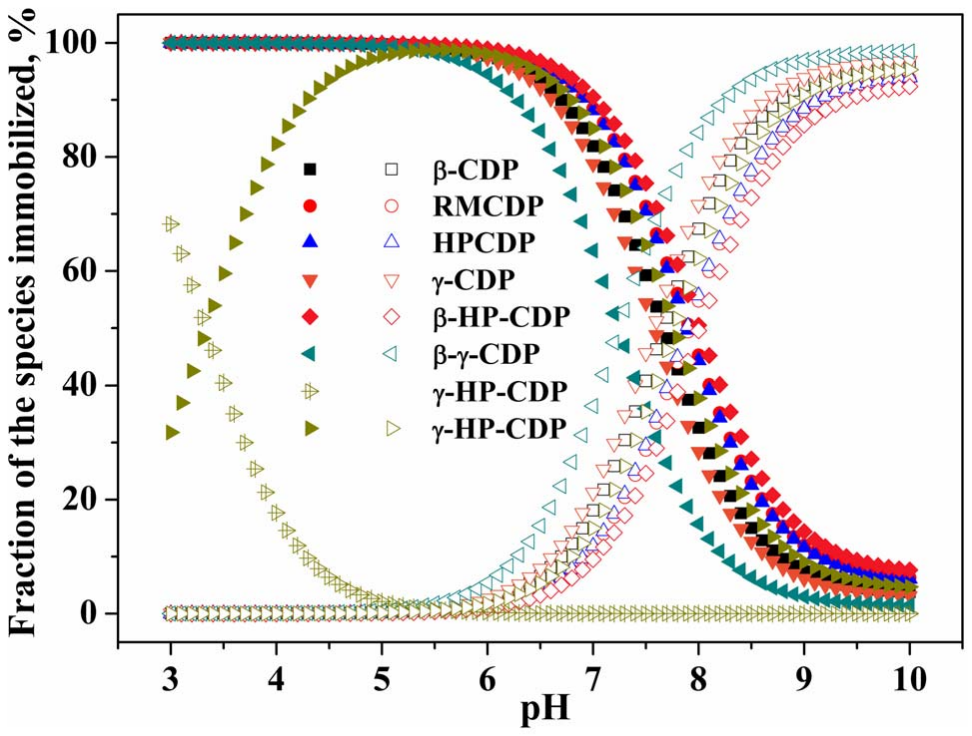
Species-specific fractions of OTC immobilized onto CDPs. The hollow with a cross, solid, and hollow scatter represent the fractions of OTCH_2_^+^, OTCH^±^ and OTC^−^, respectively.

In a recent study [47], surface coverage of the species has been highlighted for citric acid onto TiO_2_ anatase nanoparticles, and the fully deprotonated species is preferred to immobilization. In this study, immobilization potential (i.e., adsorption amounts) of the species for OTC onto the polymers was calculated on the basis of the *K*_d_ and aqueous fractions of the species. The results demonstrated the immobilization of the species was a function of pH (Figure 4). For adsorbent γ-HP-CDP, the species immobilized was OTCH_2_^+^ (9.7–68.2%) and OTCH^±^ (31.8–90.2%) at pH 3.0–4.3, OTCH^±^ (91.8–98.8%) at pH 4.4–6.7, OTCH^±^ (18.2–89.9%) and OTC^−^ (10.0–81.8%) at pH 6.8–8.5, and OTC^−^ (84.4–95.3%) at pH 8.6–10.0. For the other six adsorbents onto which immobilization of OTCH_2_^+^ was excluded, the species immobilized was OTCH^±^ (90.3–100%) at pH 3.0–6.2, OTCH^±^ (2.2–97.9%) and OTC^−^ (2.1–97.8%) at pH 6.3–9.3, and OTC^−^ (90.0–98.5%) at pH 9.4–10.0. Obviously, the differences in immobilization of the species are responsible for the apparent pH-dependent adsorption of OTC.

### Recognition of interactions of the species of OTC onto CDPs

As it is well recognized, CD inclusion is the dominant adsorption interaction for CD-based polymers toward most guest compounds [17]–[19], [38]. In most cases, high CD content corresponds to high adsorption affinity [17]–[19], [38]. In contradiction to this finding, the *K*_d_ of the species in this study (Table 2) was not correlated with CD content of the polymers that is compiled in Table 1 in our previous work [19]. Especially, γ-HP-CDP that has the second lowest CD content [19] exhibited the highest affinity to the species OTCH_2_^+^, OTCH^±^, and OTC^−^ (Table 2). Such deviation suggested that the adsorption of the species be governed by a combination of CD inclusion and other mechanisms.

The adsorption interactions were attempted to distinguish using multiple linear regression (MLR) analysis for the *K*_d_ of OTCH^±^ and OTCH^−^ with CDPs properties. CD content, cross-linking degree, swelling ratio, particle size, pore size, surface area, pore volume, and species-specific inclusion constant were respectively set as variables *x*_1_, *x*_2_, *x*_3_, *x*_4_, *x*_5_, *x*_6_, *x*_7_, and *x*_8_. The MLR analysis was performed by the stepwise method in the SPSS Statistic program (SPSS 17.0 version) (Equations 3 and 4). Collinearity between variables was excluded in terms of variance inflation factor <10. The analysis was repeated (Equations 4 and 5) when substituting variables *x*_1_ and *x*_8_ with variable *x*_9_ (denoted *x*_1_×*x*_8_). Variable *x*_9_, relating to both CD content and species-specific inclusion constant, may be indicative of effective inclusion.
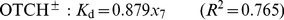
(3)

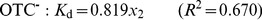
(4)

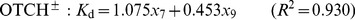
(5)


The adsorption of OTCH^±^ was correlated with pore volume and effective inclusion capacity (Equations 3 and 5). It highlights the presence of pore-filling [48], [49] and CD inclusion mechanisms for the species OTCH^±^. Comparison of standardized coefficients showed that pore-filling mechanism contributed more than CD inclusion one. It should be noted that the pore of the polymer is composed of pore channel and CD cavity. Analysis of the complex of the species with CD (Figures S4, S5) or EPI oligomers (Figure S10) demonstrated that both units could bind the species via hydrogen bonding and/or σ-π interactions. The substitution of variable *x*_9_ rendered a significant increase in the regression coefficient (*R* ^2^) (Equation 5 versus Equation 3). This improvement suggested that variable *x*_9_ was a good indicator of inclusion potential relative to variables *x*_1_ and *x*_8_. A concomitant of the regression improvement was an increase in the weight coefficient of pore volume. It is probably due to the dual roles (i.e., inclusion complexation and pore donator) of CD, both which are derived from the cavity of CD.

The adsorption of OTC^−^ was mainly related to cross-linking degree (Equation 4). High cross-linking degree commonly causes a dense network structure [19], [50]. Hydrogen bonding forces and σ-π interactions were identified for the species OTC^−^ binding to EPI oligomers (Figure S10). This correlation probably illustrates the presence of network capture mechanism. In contrast, the substitution of variable *x*_9_ didn't affect the MLR analysis of OTC^−^. This indicated an insignificant role of CD inclusion for OTC^−^, which was in agreement with the finding of its poor CD inclusion potential (Table 1).

For the other two species that are present in acidic or basic solutions (i.e., OTCH_2_^+^ and OTC^2−^), the MLR analysis wasn't performed because the *K*_d_ almost approached zero (Table 2). The poor adsorption affinity of the two species could be attributed to the fact that the adsorption sites (i.e., CD cavity) were not readily accessible for the two species, because they were difficult to diffuse to hydrophobic surfaces (Table S7). Furthermore, they could interact with hydroxyl groups in the network but in the less magnitude, as compared with the other two species. It is due to the decrease in the number of hydrogen bonding for the species following the order of OTCH^−^ (10)>OTCH^±^ (8)>both species (3) (Figure S10).

### The nature of pH-dependent adsorption of OTC

**Figure 5:**
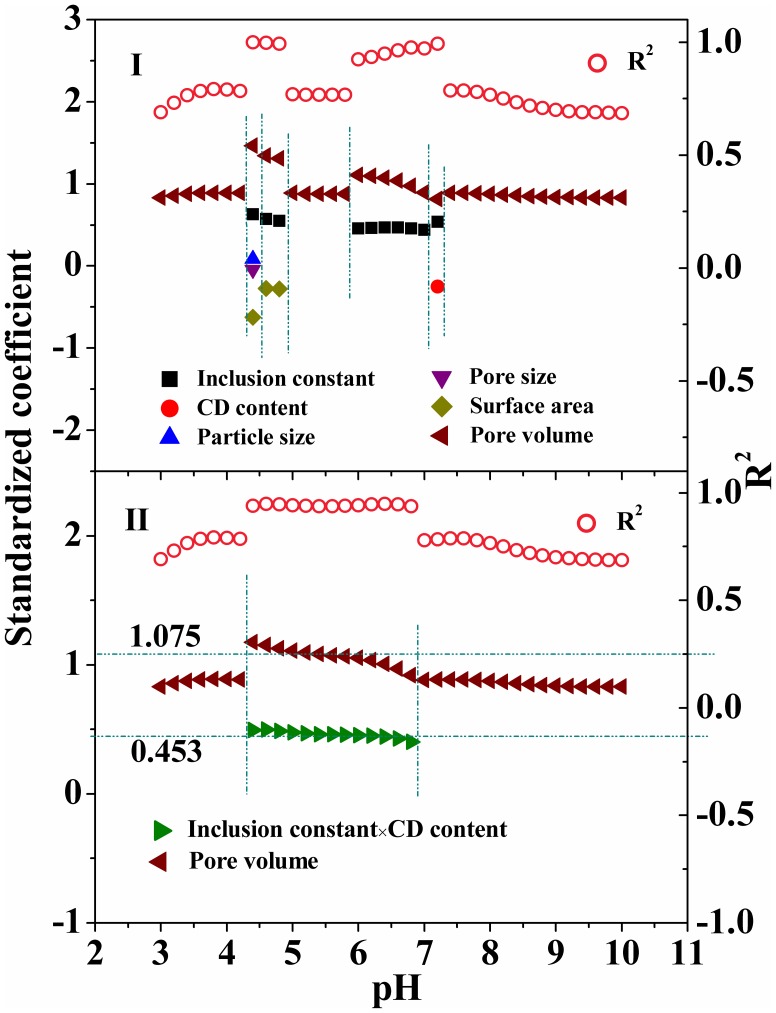
Multiple linear regression analysis of apparent adsorption of OTC at varying pH with CDPs properties. I, both CD content and inclusion constant were set as independent variables; and II, CD content×inclusion constant was set as an independent variable.

The MLR analysis of apparent *K*_d_ of OTC with CDP properties was performed, in which the apparent *K*_d_ values of OTC was calculated on the basis of Equation 2 (showed as lines in Figure 3) when pH varied from 3.0 to 10.0 with an interval of 0.2. The apparent adsorption of OTC at varying pH was correlated with one to five of all eight factors of concern, when CD content and inclusion constant were set as independent variable (Figure 5I). Surprisingly, the substitution of variable *x*_9_ reduced the number of influencing factors, only including pore volume and/or effective inclusion, while regression coefficients (*R* ^2^) were comparable (Figure 5II).

**Figure 6:**
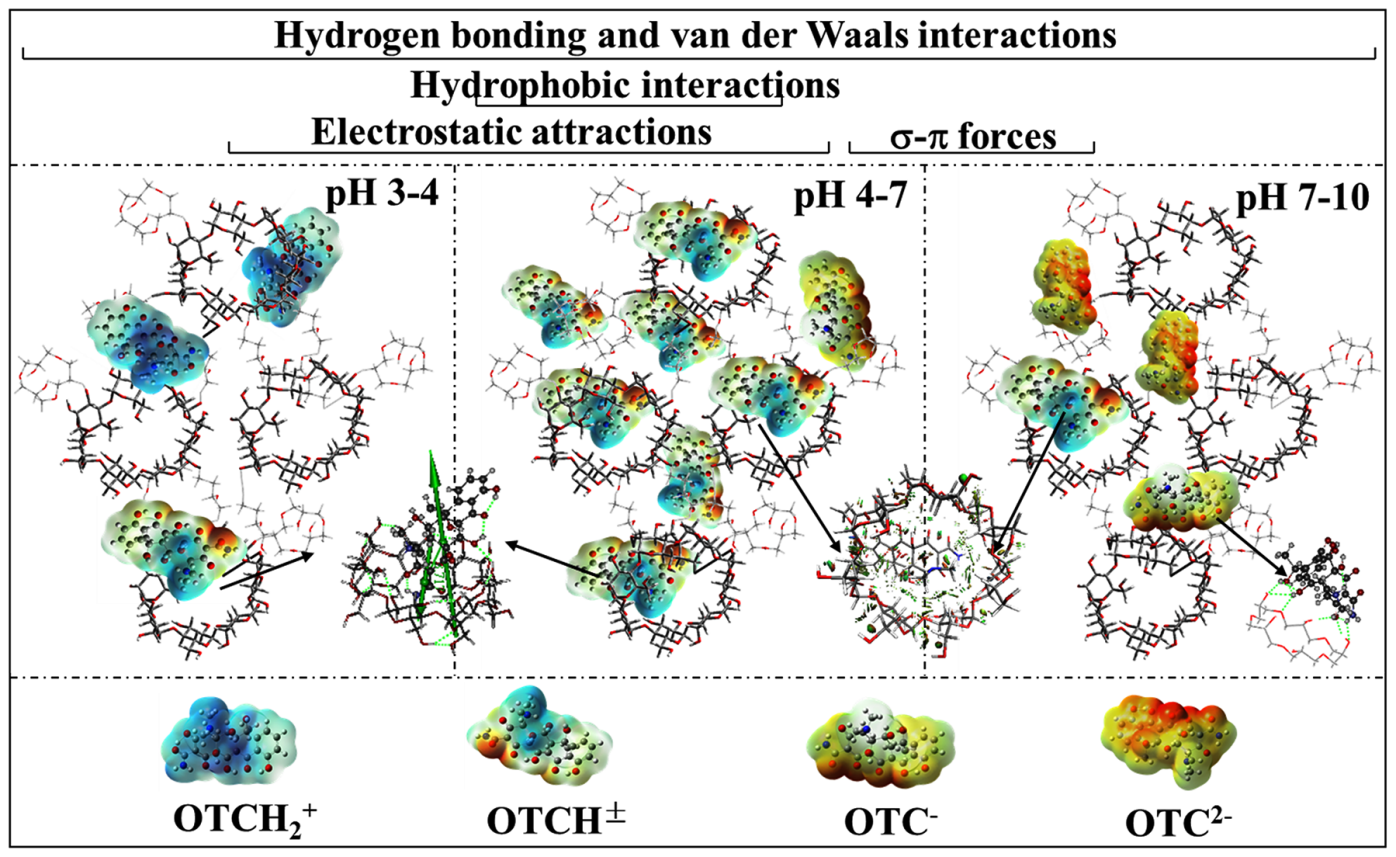
Species-specific interactions involved in the adsorption of OTC at varying pH onto CDPs.

A combination of the MLR analysis for OTC (Figure 5II) and molecular recognition in adsorption of the species (Equations 3–5) may illustrate pH-dependent adsorption of OTC onto CDPs (Figure 6). Specifically, the term *K*_d_ correlates with pore volume at pH 3.0–4.2, where OTCH^±^ and OTCH_2_^+^ prevail and are adsorbed via pore-filling mechanism. As the solution pH rises up to 6.8, pore volume and effective inclusion capacity become the influencing factors corresponding to pore-filling and CD inclusion mechanisms, respectively. It is reasonable because OTCH^±^ is the predominant species in the range of pH. However, the standardized coefficients of both influencing factors (Figure 5II) gradually deviate from those in Equation 5 with pH increasing. It is probably due to an increase in the fraction of OTC^−^ over pH. Surprisingly, the term pore volume is the sole influencing factor at pH 7.0–10.0, indicating the presence of pore-filling mechanism. It is in contradiction to the fact that the species OTC^−^ predominates at pH 8.0–9.0 and its adsorption proceeds via network capture mechanism. This may be due to a combination of pore-filling, CD inclusion and network capture mechanisms, associated with the coexistence of two or three species in this pH range.

### Selection of adsorbent to separate OTC from water

**Figure 7:**
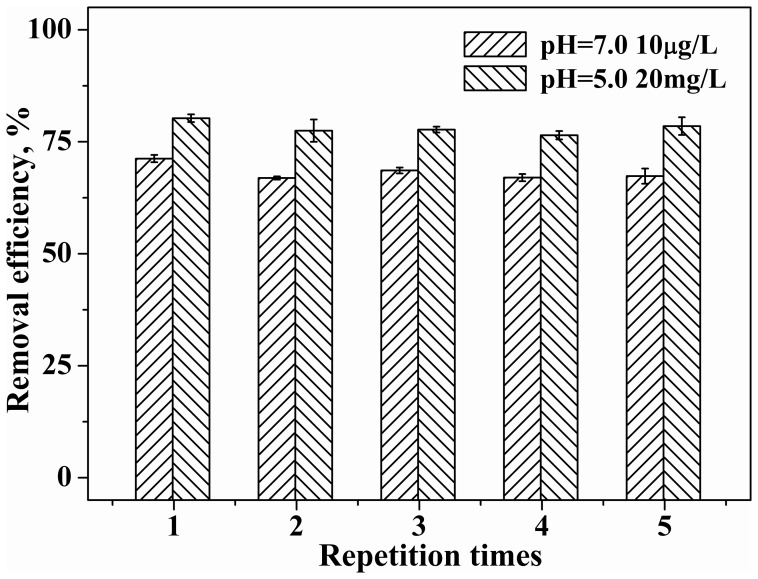
Removal efficiency of OTC by adsorbent γ-HP-CDP.

The selection of the polymers may be determined on the basis of the understanding of the nature of pH-dependent adsorption of OTC (Figure 5II). Adsorbent γ-HP-CDP with advantages of high pore volume and efficient inclusion potential was selected to separate and remove OTC from simulated wastewater (pH 5.0) and natural water (pH 7.0) (Text S4). The adsorbent removed 80% of OTC at 20 mg/L (pH 5.0) (Figure 7). In contrast, other conventional natural and synthetic adsorbents are observed to exhibit similar or lower adsorption efficiency for OTC, such as activated carbon (ca. 68%) [7], [51], multi-wall carbon nanotubes (ca. 70%) [52], and aluminum oxide (44%) [53]. Furthermore, the polymer exhibits a removal efficiency of above 70% for the antibiotic even at environmentally relevant levels (Figure 7).

In addition, ubiquitously existing Ca^2+^ (e.g., at 150 and 50 mg/L), Mg^2+^ (e.g., at 50 and 8 mg/L) and DOM (e.g., at 10 mg/L) that are natural water constituents and usually are indicative of environmental matrix effects did not deteriorate removal efficiency of OTC in wastewater (pH = 5.0) (Table 3). This is consistent with the findings of other studies about the removal of neutral pollutants by cyclodextrin materials [19], [54]. In contrast, many works have showed negative effects of cations (Na^+^, Ca^2+^, Mg^2+^) and DOM on removal rate of tetracyclines by conventional adsorbents (e.g., Na-montmorillonite [55], humic acids [56], graphene oxide [57], CNTs [58], kaolinite [59], and clays [46]). In some cases, the decrease in removal rate approaches about 50–90% [58], [59].

**Table 3:** Effect of coexisting components on removal efficiency of OTC by γ-HP-CDP at pH 5.0.

	Removal efficiency, %
OTC, 20 mg/L (control)	80.7±0.9
Ca^2+^, 150 mg/L	80.5±0.2
Ca^2+^, 50 mg/L	81.3±0.4
Mg^2+^, 50 mg/L	80.4±0.1
Mg^2+^, 8 mg/L	80.8±0.8
DOM, 10 mg/L	80.7±1.0

## Conclusions

The finding of the species-specific adsorption reveals the pH-dependent adsorption behavior of antibiotic oxytetracycline onto adsorbent CDPs. The adsorption sites of the polymer such as CD cavity, pore channel, and network are accessible for the species to different extents, associated with the coordination of a variety of weak interactions (e.g., hydrogen bonding, hydrophobic interactions, van der Waals forces and electrostatic interactions). The species OTCH^±^ commonly has high adsorption affinity via pore-filling and CD inclusion mechanisms, OTC^−^ exhibits moderate affinity via network capture mechanism and the other two species are poorly adsorbed due to the limiting accessibility of the inclusion sites as well as other adsorption sites. The resulting immobilization of the species onto CDPs is determined by the *K*_d_ and fraction of the species in solution, representing a function of pH. This may be the cause for the pH-dependence of adsorption of OTC. Moreover, the mathematical equations are derived to quantitatively relate adsorption affinity of OTC at varying pH to adsorbent properties. These relationships, together with the molecular recognition into adsorption of the species, provide theoretical guidelines for the selection of CDPs to remove the antibiotic. Additionally, this study indicates that CDPs are promising adsorbents for IOCs removal, taking advantage of discernible adsorption interactions, high removal efficiency, and strong resistance to the interference of coexisting natural components.

## Supporting Information

Text S1 **Adsorption kinetics and isotherms of OTC.**
(DOC)

Text S2 **Determination of the stability of OTC.**
(DOC)

Text S3 **LINGO optimization program of species-specific interaction constant.**
(DOC)

Text S4 **Removal of OTC from simulated waters.**
(DOC)

Table S1 **Related information of cyclodextrin.**
(DOC)

Table S2 **Inclusion complexation of CD with OTC at varying pH.**
(DOC)

Table S3 **Linear correlation between** ***K*** **_C_ values calculated and experimental.**
(DOC)

Table S4 **^1^H chemical shifts (ppm) of OTC, CD and CD-OTC protons.**
(DOC)

Table S5 **Intermolecular interactions of CD and the species.**
(DOC)

Table S6 **Parameters of the pseudo -first and -second order model.**
(DOC)

Table S7 **Correlation coefficients (** ***R*** **^2^) of the moving boundary model.**
(DOC)

Table S8 **Adsorption parameters of OTC fitted to Langmuir model.**
(DOC)

Table S9 **Adsorption parameters of OTC fitted to Freundlich model.**
(DOC)

Table S10 **Linear correlation between** ***K*** **_d_ values calculated and experimental.**
(DOC)

Figure S1 **UV-Vis spectra of CD-OTC complexes at varying pH.**
(TIF)

Figure S2 **FTIR spectra of CD, OTC and CD-OTC complexes.**
(TIF)

Figure S3 **^1^H NMR spectra of OTC, CD and CD-OTC complexes (dissolved in D_2_O).** HOD at 4.617 ppm was taken as a reference.
(TIF)

Figure S4 **Molecular docking of CD with the species.** The green dotted line represents hydrogen bonding.
(TIF)

Figure S5 **Qualitative comparison of intermolecular interactions between CD and the species.** The blue, green, and red isosurfaces are indicative of hydrogen bonding, van der Waals forces, and repulsion interactions, respectively.
(TIF)

Figure S6 **Adsorption kinetics of OTC onto CDPs at pH 7.0.**
(TIF)

Figure S7 **Pseudo-second order model for adsorption of OTC onto CDPs (pH 7.0).**
(TIF)

Figure S8 **Adsorption isotherms of OTC onto CDPs at varying pH.**
(TIF)

Figure S9 **FTIR spectra of CDP adsorbing OTC at respective pH 5.0, 7.0 and 9.0.**
(TIF)

Figure S10 **Molecular docking of the EPI oligomer with the species.** The green dotted line represents hydrogen bonding and the orange line represents σ-π interaction. The numbers in parentheses represent ring-opened, tricyclo-, pentacyclo-, and heptacyclo-forms, respectively.
(TIF)
