# Supplementary material for: Molecular Insights into the pH-Dependent Adsorption and Removal of Ionizable Antibiotic Oxytetracycline by Adsorbent Cyclodextrin Polymers
Source: PLoS One. 2014 Jan 21;9(1):e86228. doi: 10.1371/journal.pone.0086228 (PMC3897700; doi:10.1371/journal.pone.0086228)
Supplement: Text S1 — Adsorption kinetics and isotherms of OTC. (DOC) [file pone.0086228.s001.doc]

**Text S1.** Adsorption kinetics and isotherms of OTC.

Adsorption kinetic experiments were conducted at pH 7.0. Both pseudo-first () and pseudo-second order () models were fitted. Where *K*_1_ (1/min) and *K*_2_ (Kg/(mg·min) are rate constants of pseudo-first and pseudo-second order models, respectively; *Q*_e_ () (mg/Kg) and *Q_s_*_,t_ (mg/g) are adsorption amounts at equilibrium and time *t* (min), respectively; *C*_0_ (mg/L) is initial concentration of OTC; *C*_e_ (mg/L) is solution concentration of OTC at equilibrium; *V* (mL) is volume of solution; *m* (Kg) is mass of CDP used; *F* is adsorption fraction (*Q*_s,t_/*Q*_e_); and *k* is adsorption rate constant. The rate controlling step was determined according to the moving boundary model . Liquid film diffusion (), intraparticle diffusion () and chemical interaction () were compared on basis of the corresponding correlation coefficients. The process with the highest coefficient was considered as the rate-controlling step.

Data for adsorption isotherm was fitted to both Langmuir () and Freundlich () models. Where *Q*_m_ (mg/Kg) is adsorption capacity; *K*_L_ (L/mg) and *K*_F_ (mg^1-1/n^L^1/n^Kg^-1^) are adsorption constants from Langmuir and Freundlich models, respectively; and *n* is Freundlich heterogeneity factor.

The adsorption distribution coefficients (, L/Kg) of different initial concentrations were calculated. Then the average *K*_d_ values were obtained to compare the adsorption affinity due to their dimensionless.

Reference

1. Boyd GE, Myers LS, Adamson AW (1947) The exchange adsorption of ions from aqueous solutions by organic zeolites .3. performance of deep adsorbent beds under non-equilibrium conditions. Journal of the American Chemical Society 69: 2849-2859.
