# Supplementary material for: Molecular Insights into the pH-Dependent Adsorption and Removal of Ionizable Antibiotic Oxytetracycline by Adsorbent Cyclodextrin Polymers
Source: PLoS One. 2014 Jan 21;9(1):e86228. doi: 10.1371/journal.pone.0086228 (PMC3897700; doi:10.1371/journal.pone.0086228)
Supplement: Text S2 — Determination of the stability of OTC. (DOC) [file pone.0086228.s002.doc]

**Text S2.** Determination of the stability of OTC.

The stability of OTC was determined by HPLC in neutral solution at the temperature of 4, 15, 20, 25, and 35 °C, respectively. Solutions of OTC adjusted to pH 2.0 (30 °C) and 10.0 (25 °C) were also tested, respectively. Results were shown in the figures below. In neutral solution, OTC was stable within 24 hours below 15 °C. However, OTC decreased fast as the temperature went up. These results were in accord with the previous report that OTC was unstable in water solution, especially at high temperatures . In the solution of pH 10.0, OTC kept unchanged at 25 °C within the initial two hours. In contrast, in the solutions acidified to pH 2.0, the antibiotic exhibited good stability even at 35 °C. Under all experiments where transformation products of OTC weren’t detected, the stability of the antibiotic was ensured.

| 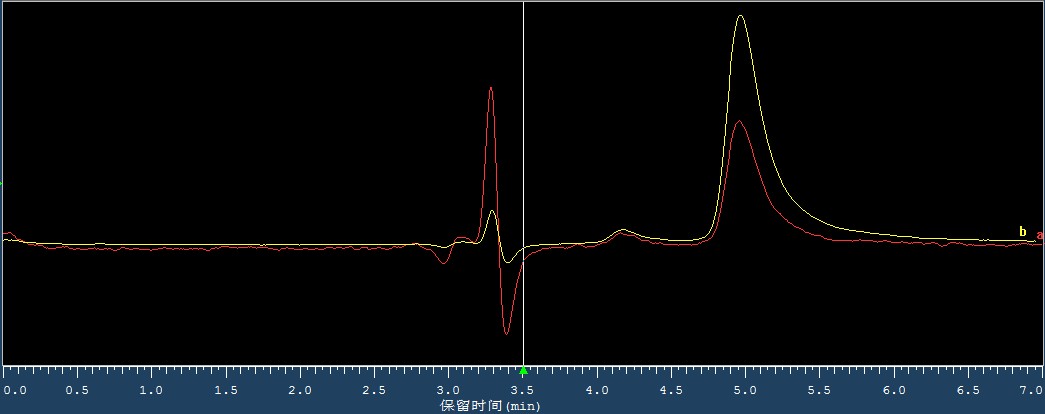 |
| --- |

Reference

2. Rose MD, Bygrave J, Farrington WHH, Shearer G (1996) The effect of cooking on veterinary drug residues in food .4. Oxytetracycline. Food Additives and Contaminants 13: 275-286.
