# Supplementary material for: Molecular Insights into the pH-Dependent Adsorption and Removal of Ionizable Antibiotic Oxytetracycline by Adsorbent Cyclodextrin Polymers
Source: PLoS One. 2014 Jan 21;9(1):e86228. doi: 10.1371/journal.pone.0086228 (PMC3897700; doi:10.1371/journal.pone.0086228)
Supplement: Text S3 — LINGO optimization program of species-specific interaction constant. (DOC) [file pone.0086228.s003.doc]

**Text S3.** LINGO optimization program of species-specific interaction constant.

At a given pH, mass fractions of the species, *α*^i^ (i=+, ±, -, 2-), can be calculated using the following equations, respectively.

(S1)

(S2)

(S3)

(S4)

where p*K*_a1_, p*K*_a2_ and p*K*_a3_ are 3.27, 7.32 and 9.11, respectively.

The obtained *α*^i^ at certain pH and corresponding experimental *K* were substituted into the optimization program in the figure below. Click “solve” and interaction constants of the species would be output.


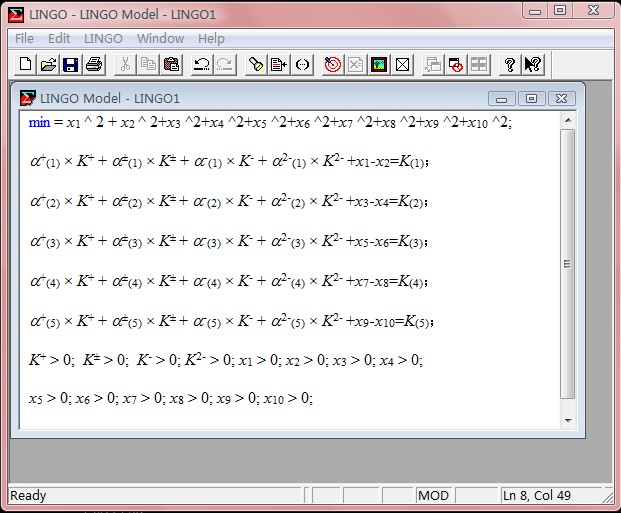


Solve
