# Supplementary material for: Molecular Insights into the pH-Dependent Adsorption and Removal of Ionizable Antibiotic Oxytetracycline by Adsorbent Cyclodextrin Polymers
Source: PLoS One. 2014 Jan 21;9(1):e86228. doi: 10.1371/journal.pone.0086228 (PMC3897700; doi:10.1371/journal.pone.0086228)
Supplement: Text S4 — Removal of OTC from simulated waters. (DOC) [file pone.0086228.s004.doc]

**Text S4.** Removal of OTC from simulated waters.

Removal of OTC by γ-HP-CDP from simulated waters was conducted in 40-ml glass centrifuge tubes at 25 °C. A solid-liquid ratio was 1:50. Initial concentrations of OTC were 10 μg/L and 20 mg/L, often encountered in natural aquatic systems and pharmaceutical wastewaters , respectively. The solutions were adjusted to pH 7.0 (for 10 μg/L OTC) and 5.0 (for 20 mg/L OTC) using 1-mol/L HCl, respectively. Besides, the effect of Ca^2+^, Mg^2+^ and DOM on removal efficiency of OTC (20 mg/L) by γ-HP-CDP at pH 5.0 was also respectively inspected. The investigated concentrations of Ca^2+^, Mg^2+^ and DOM were 150 and 50 mg/L, 50 and 8 mg/L, and 10mg/L, respectively, which were common levels in wastewaters and natural waters. All the samples were shaken by a rotary shaker (180 rpm) in the dark. After 30 min, phase separation was performed and the supernatant was acidified to pH ca. 2.5. OTC in the supernatant was determined by LC/MS/MS (Agilent 1200 series) and HPLC, respectively. The applied conditions for LC/MS/MS: the stationary phase was an Agilent Eclipse Plus C18 column (2.1 × 150 mm, 5 μm); the mobile phase comprised 30% acetonitrile and 70% formic acid solution (0.2% v); the flow rate was set at 0.25 ml/min; the column temperature was 40°C. Mass spectra were performed in positive ESI MRM mode; delta EMV (+) was set at 400 and collision energy was 30V.

The separated adsorbent was transferred into 30 ml of the mobile phase of HPLC (Acetonitrile: NaH_2_PO_4_-H_3_PO_4_ buffer solution = 22%:78%, pH=2.30) and shaken for 30 min. Thereafter, the solution was centrifuged. This procedure was conducted three times to ensure complete extraction. Finally, the adsorbent was washed with water and dried for 10 h at 60 °C. The reuse experiment of γ-HP-CDP was carried out five times to assess the regeneration ability.

Reference

3. Luo Y, Xu L, Rysz M, Wang YQ, Zhang H, et al. (2011) Occurrence and transport of tetracycline, sulfonamide, quinolone, and macrolide antibiotics in the Haihe River Basin, China. Environmental Science & Technology 45: 1827-1833.

4. Chen YS, Zhang HB, Luo YM, Song J (2012) Occurrence and dissipation of veterinary antibiotics in two typical swine wastewater treatment systems in east China. Environmental Monitoring and Assessment 184: 2205-2217.

5. Homem V, Santos L (2011) Degradation and removal methods of antibiotics from aqueous matrices - A review. Journal of Environmental Management 92: 2304-2347.
