# Supplementary material for: Molecular Insights into the pH-Dependent Adsorption and Removal of Ionizable Antibiotic Oxytetracycline by Adsorbent Cyclodextrin Polymers
Source: PLoS One. 2014 Jan 21;9(1):e86228. doi: 10.1371/journal.pone.0086228 (PMC3897700; doi:10.1371/journal.pone.0086228)
Supplement: Table S1 — Related information of cyclodextrin. (DOC) [file pone.0086228.s005.doc]

**Table S1. Related information of cyclodextrin.**

| Abbr. *^a^* | Cyclodextrion | D.S. *^b^* | M.W. *^c^* | Source |
| --- | --- | --- | --- | --- |
| β-CD | β-cyclodextrin | 0.0 | 1135.00 | Tianjin Bodi Chemical Co., Ltd. |
| HPCD | Hydroxypropyl-β-cyclodextrin | 6.4 | 1506.20 | Xinda Fine Chemical Co., Ltd. |
| RMCD | Randomly methylated-β-cyclodextrin | 5.1 | 1206.40 | Xinda Fine Chemical Co., Ltd. |
| γ-CD | γ-cyclodextrin | 0.0 | 1297.00 | Wuhan Galaxy Chemical Co., Ltd |

***^a^*** Abbreviation ***^b^*** Degree of Substitution ***^c^*** Molecular Weight
