# Supplementary material for: Molecular Insights into the pH-Dependent Adsorption and Removal of Ionizable Antibiotic Oxytetracycline by Adsorbent Cyclodextrin Polymers
Source: PLoS One. 2014 Jan 21;9(1):e86228. doi: 10.1371/journal.pone.0086228 (PMC3897700; doi:10.1371/journal.pone.0086228)
Supplement: Table S2 — Inclusion complexation of CD with OTC at varying pH. (DOC) [file pone.0086228.s006.doc]

**Table S2.** Inclusion complexation of CD with OTC at varying pH.

|  | pH | Inclusion ratio, *n* | Inclusion constant, *K* (L/mol) | *R*^2^ |
| --- | --- | --- | --- | --- |
| β-CD | 4.20 | 1:1 | 456.05±30.53 | 0.96 |
|  | 4.75 | 1:1 | 937.71±45.97 | 0.99 |
|  | 6.50 | 1:1 | 536.82±13.65 | 0.99 |
|  | 7.16 | 1:1 | 72.56±9.66 | 0.99 |
|  | 8.50 | 1:1 | 90.54±12.21 | 0.97 |
| RMCD | 4.17 | 1:1 | 379.50±19.33 | 0.99 |
|  | 4.77 | 1:1 | 135.18±5.44 | 0.98 |
|  | 6.36 | 1:1 | 64.24±4.89 | 0.98 |
|  | 6.85 | 1:1 | 114.74±15.23 | 0.96 |
|  | 8.69 | 1:1 | 327.73±35.64 | 0.97 |
| HPCD | 4.23 | 1:1 | 96.14±17.89 | 1.00 |
|  | 4.85 | 1:1 | 312.26±34.23 | 0.99 |
|  | 6.36 | 1:1 | 317.71±27.99 | 0.98 |
|  | 7.84 | 1:1 | 372.82±12.33 | 0.99 |
|  | 9.30 | 1:1 | 721.00±5.39 | 0.98 |
| γ-CD | 4.33 | 1:1 | 58.27±4.34 | 0.98 |
|  | 4.80 | 1:1 | 141.08±18.22 | 0.98 |
|  | 6.42 | 1:1 | 206.66±25.39 | 1.00 |
|  | 7.43 | 1:1 | 20.06±3.96 | 0.99 |
|  | 9.23 | 1:1 | 274.02±13.99 | 0.96 |
