# Supplementary material for: Molecular Insights into the pH-Dependent Adsorption and Removal of Ionizable Antibiotic Oxytetracycline by Adsorbent Cyclodextrin Polymers
Source: PLoS One. 2014 Jan 21;9(1):e86228. doi: 10.1371/journal.pone.0086228 (PMC3897700; doi:10.1371/journal.pone.0086228)
Supplement: Table S3 — Linear correlation between K C values calculated and experimental. (DOC) [file pone.0086228.s007.doc]

**Table S3.** Linear correlation between *K*_C_ values calculated and experimental.

|  | Equation | *R*^2^ | P |
| --- | --- | --- | --- |
| β-CD | *y*^a^=0.79*x*^b^ | 0.74 | < 0.05 |
| RMCD | *y*=1.00*x* | 1.00 | < 0.01 |
| HPCD | *y*=1.01*x* | 0.88 | < 0.01 |
| γ-CD | *y*=0.89*x* | 0.87 | < 0.01 |

^a^ the values were calculated on the basis of Equation 2 into which the *K*_C_ and fraction of the species were substituted

^b^ the values were obtained from spectroscopic titration experiments.
