# Supplementary material for: Molecular Insights into the pH-Dependent Adsorption and Removal of Ionizable Antibiotic Oxytetracycline by Adsorbent Cyclodextrin Polymers
Source: PLoS One. 2014 Jan 21;9(1):e86228. doi: 10.1371/journal.pone.0086228 (PMC3897700; doi:10.1371/journal.pone.0086228)
Supplement: Table S4 — 1H chemical shifts (ppm) of OTC, CD and CD-OTC protons. (DOC) [file pone.0086228.s008.doc]

**Table S4.** ^1^H chemical shifts (ppm) of OTC, CD and CD-OTC protons.^a^

|  | H1 | H2 | H3 | H4 | H5 | H6 | C4-NH(CH3)2 | C6-CH3 |
| --- | --- | --- | --- | --- | --- | --- | --- | --- |
| OTC |  |  |  |  |  |  | 2.686 | 1.601 |
| β-CD | 4.842 | 3.420 | 3.741 | 3.357 | 3.651 | 3.643 |  |  |
| β-CD-OTC | 4.879 | 3.458 | 3.771 | 3.393 | 3.685 | 3.677 | 2.698 | 1.635 |
| RMCD | 4.980 | 3.374 | 3.775 | 3.207 | 3.686 | 3.469 |  |  |
| RMCD-OTC | 4.977 | 3.373 | 3.824 | 3.206 | 3.682 | 3.463 | 2.692 | 1.643 |
| HPCD | 4.992 | 3.418 | 3.826 | 3.283 | 3.673 | 3.534 |  |  |
| HPCD-OTC | 4.985 | 3.417 | 3.830 | 3.287 | 3.672 | 3.531 | 2.716 | 1.648 |
| γ-CD | 4.920 | 3.464 | 3.745 | 3.424 | 3.681 | 3.661 |  |  |
| γ-CD-OTC | 4.921 | 3.455 | 3.745 | 3.401 | 3.680 | -^b^ | 2.731 | 1.639 |

^a^ Reference: HOD-4.617 ppm

^b^ no value given by the NMR test
