# Supplementary material for: Molecular Insights into the pH-Dependent Adsorption and Removal of Ionizable Antibiotic Oxytetracycline by Adsorbent Cyclodextrin Polymers
Source: PLoS One. 2014 Jan 21;9(1):e86228. doi: 10.1371/journal.pone.0086228 (PMC3897700; doi:10.1371/journal.pone.0086228)
Supplement: Table S5 — Intermolecular interactions of CD and the species. (DOC) [file pone.0086228.s009.doc]

**Table S5. Intermolecular interactions of CD and the species.**

|  |  | OTCH_2_^+^ | OTCH^±^ | OTC^-^ | OTC^2-^ |
| --- | --- | --- | --- | --- | --- |
| β-CD | E_t_^a^ | -156.03 | -129.19 | -260.88 | -267.36 |
|  | E_v_^b^ | -49.79 | -51.29 | -28.49 | -39.72 |
|  | E_e_^c^ | -106.24 | -77.90 | -232.40 | -227.64 |
| RMCD | E_t_ | -118.33 | -127.95 | -191.93 | -258.96 |
|  | E_v_ | -51.76 | -52.29 | -56.83 | -28.85 |
|  | E_e_ | -66.57 | -75.67 | -135.10 | -230.11 |
| HPCD | E_t_ | -146.43 | -131.06 | -236.51 | -321.51 |
|  | E_v_ | -49.45 | -37.99 | -38.86 | -50.43 |
|  | E_e_ | -96.98 | -93.07 | -197.65 | -271.08 |
| γ-CD | E_t_ | -110.793 | -183.26 | -234.47 | -290.55 |
|  | E_v_ | -54.36 | -36.46 | -33.89 | -40.59 |
|  | E_e_ | -58.43 | -146.90 | -200.57 | -249.96 |

^a^ total interaction energy, kcal/mol

^b^ van der waals energy, kcal/mol

^c^ electrostatic energy, kcal/mol
