# Supplementary material for: Molecular Insights into the pH-Dependent Adsorption and Removal of Ionizable Antibiotic Oxytetracycline by Adsorbent Cyclodextrin Polymers
Source: PLoS One. 2014 Jan 21;9(1):e86228. doi: 10.1371/journal.pone.0086228 (PMC3897700; doi:10.1371/journal.pone.0086228)
Supplement: Table S6 — Parameters of the pseudo -first and -second order model. (DOC) [file pone.0086228.s010.doc]

**Table S6.** Parameters of the pseudo -first and -second order model.^a^

|  | pseudo-first order model | | | pseudo-second order model | | |
| --- | --- | --- | --- | --- | --- | --- |
|  | *K*_1_,1/min | *Q*_e_, mg/g | *R*^2^ | *K*_2_, g/(mg.min) | *Q*_e_, mg/g | *R*^2^ |
| β-CDP | 0.2870 | 0.1870 | 0.690 | 0.0026 | 0.2039 | 0.999 |
| RMCDP | 0.1693 | 0.4695 | 0.840 | 0.0402 | 0.4925 | 0.999 |
| HPCDP | 0.4749 | 0.1019 | 0.745 | 0.1201 | 0.5255 | 1.000 |
| γ-CDP | 0.4617 | 0.2372 | 0.783 | 0.0391 | 0.4964 | 1.000 |
| β-HP-CDP | 0.4852 | 0.2981 | 0.774 | 0.0426 | 0.5213 | 1.000 |
| β-γ-CDP | 0.4235 | 0.3040 | 0.678 | 0.0224 | 0.4704 | 0.998 |
| γ-HP-CDP | 0.5323 | 0.2303 | 0.822 | 0.1002 | 0.5367 | 0.999 |

^a^ *K*_1_ and *K*_2_ are rate constants of first and pseudo-second order models, respectively; and *Q*_e_ is adsorption amount at equilibrium.
