# Supplementary material for: Molecular Insights into the pH-Dependent Adsorption and Removal of Ionizable Antibiotic Oxytetracycline by Adsorbent Cyclodextrin Polymers
Source: PLoS One. 2014 Jan 21;9(1):e86228. doi: 10.1371/journal.pone.0086228 (PMC3897700; doi:10.1371/journal.pone.0086228)
Supplement: Table S7 — Correlation coefficients ( R 2) of the moving boundary model. (DOC) [file pone.0086228.s011.doc]

**Table S7.** Correlation coefficients (*R*^2^) of the moving boundary model.

|  | Liquid film diffusion | Intraparticle diffusion | Chemical interaction |
| --- | --- | --- | --- |
| β-CDP | 0.85 | 0.78 | 0.79 |
| RMCDP | 0.30 | 0.50 | 0.47 |
| HPCDP | 0.94 | 0.80 | 0.87 |
| γ-CDP | 0.78 | 0.68 | 0.66 |
| β-HP-CDP | 0.82 | 0.73 | 0.72 |
| β-γ-CDP | 0.77 | 0.84 | 0.83 |
| γ-HP-CDP | 0.32 | 0.22 | 0.22 |
