# Supplementary material for: Molecular Insights into the pH-Dependent Adsorption and Removal of Ionizable Antibiotic Oxytetracycline by Adsorbent Cyclodextrin Polymers
Source: PLoS One. 2014 Jan 21;9(1):e86228. doi: 10.1371/journal.pone.0086228 (PMC3897700; doi:10.1371/journal.pone.0086228)
Supplement: Table S8 — Adsorption parameters of OTC fitted to Langmuir model. (DOC) [file pone.0086228.s012.doc]

**Table S8. Adsorption parameters of OTC fitted to Langmuir model.**

|  | pH | Adsorption constant  *K*_L_ (L/mg) | Adsorption capactiy  *Q*_m_ (mg/Kg) | *R*^2^ |
| --- | --- | --- | --- | --- |
| β-CDP | 6.41 | -^a^ | - | - |
|  | 6.60 | - | - | - |
|  | 7.73 | 0.03±0.03 | (1.29±0.71) × 10^3^ | 0.94 |
|  | 8.34 | - | - | - |
|  | 9.61 | 0.00±0.01 | (1.71±5.04) × 10^3^ | 0.93 |
| RMCDP | 4.88 | 0.06±0.06 | (4.25±3.44) × 10^3^ | 0.96 |
|  | 5.50 | 0.06±0.03 | (5.29±2.18) × 10^3^ | 0.99 |
|  | 6.75 | 0.23±0.09 | (1.61±0.36) × 10^3^ | 0.96 |
|  | 8.10 | 0.05±0.06 | (2.49±2.29) × 10^3^ | 0.92 |
|  | 8.90 | 0.01±0.01 | (1.90±1.27) × 10^3^ | 0.99 |
| HPCDP | 4.65 | 0.09±0.02 | (1.11±0.14) × 10^3^ | 0.99 |
|  | 5.35 | 0.15±0.07 | (4.01±1.36) × 10^3^ | 0.99 |
|  | 6.79 | 0.08±0.09 | (8.45±8.71) × 10^3^ | 0.97 |
|  | 8.09 | 0.47±0.15 | (0.92±0.09) × 10^3^ | 0.95 |
|  | 9.72 | 0.04±0.02 | (0.30±0.11) × 10^3^ | 0.96 |
| γ-CDP | 4.96 | 0.04±0.02 | (2.16±0.68) × 10^3^ | 0.99 |
|  | 6.00 | - | - | - |
|  | 8.98 | 0.01±0.02 | (3.12±3.90) × 10^3^ | 0.96 |
|  | 9.30 | 0.01±0.01 | (3.29±3.40) × 10^3^ | 0.98 |
|  | 10.11 | - | - | - |
| β-HP-CDP | 5.76 | - | - | - |
|  | 6.57 | 0.25±0.08 | (3.76±0.98) × 10^3^ | 0.99 |
|  | 6.88 | 0.27±0.08 | (1.94±0.34) × 10^3^ | 0.98 |
|  | 8.13 | 0.16±0.10 | (1.48±0.52) × 10^3^ | 0.94 |
|  | 9.51 | 0.01±0.01 | (0.83±0.76) × 10^3^ | 0.97 |
| β-γ-CDP | 5.06 | 0.03±0.03 | (2.81±1.95) × 10^3^ | 0.95 |
|  | 6.40 | - | - | - |
|  | 7.57 | 0.08±0.03 | (2.41±0.61) × 10^3^ | 0.99 |
|  | 8.63 | 0.01±0.02 | (8.35±13.09) × 10^3^ | 0.98 |
|  | 9.81 | 0.01±0.00 | (0.84±0.29) × 10^3^ | 0.99 |
| γ-HP-CDP | 5.80 | 0.14±0.12 | (7.79±5.95) × 10^3^ | 0.97 |
|  | 6.46 | 0.12±0.03 | (6.61±1.37) × 10^3^ | 1.00 |
|  | 7.24 | 0.40±0.18 | (3.03±0.99) × 10^3^ | 0.97 |
|  | 9.30 | 0.24±0.10 | (0.35±0.05) × 10^3^ | 0.90 |
|  | 10.07 | 0.02±0.02 | (0.50±0.55) × 10^3^ | 0.86 |

^a^ no applicable to the model
