# Supplementary material for: Molecular Insights into the pH-Dependent Adsorption and Removal of Ionizable Antibiotic Oxytetracycline by Adsorbent Cyclodextrin Polymers
Source: PLoS One. 2014 Jan 21;9(1):e86228. doi: 10.1371/journal.pone.0086228 (PMC3897700; doi:10.1371/journal.pone.0086228)
Supplement: Table S9 — Adsorption parameters of OTC fitted to Freundlich model. (DOC) [file pone.0086228.s013.doc]

**Table S9.** Adsorption parameters of OTC fitted to Freundlich model.

|  | pH | Heterogeneity factor  *n* | Adsorption constant  *K*_F_ | *R*^2^ |
| --- | --- | --- | --- | --- |
| β-CDP | 6.41 | 0.88 (0.82, 0.96) | 67.14 (58.65, 76.87) | 0.97 |
|  | 6.60 | 0.91 (0.85, 0.97) | 44.96 (39.86, 50.71) | 0.98 |
|  | 7.73 | 1.40 (1.26, 1.58) | 57.53 (48.82, 67.79) | 0.94 |
|  | 8.34 | 0.83 (0.80, 0.87) | 16.26 (14.76, 17.91) | 0.99 |
|  | 9.61 | 1.17 (1.11, 1.23) | 9.38 (8.40, 10.46) | 0.98 |
| RMCDP | 4.88 | 0.86 (0.80, 0.92) | 183.02 (168.24, 199.10) | 0.98 |
|  | 5.50 | 0.84 (0.76, 0.92) | 241.44 (216.13, 269.70) | 0.96 |
|  | 6.75 | 1.03 (0.93, 1.14) | 217.92 (195.17, 243.32) | 0.95 |
|  | 8.10 | 0.94 (0.88, 1.02) | 96.92 (86.13, 109.05) | 0.97 |
|  | 8.90 | 0.87 (0.82, 0.92) | 12.28 (10.72, 14.07) | 0.98 |
| HPCDP | 4.65 | 1.07 (0.97, 1.20) | 65.92 (55.81, 77.86) | 0.95 |
|  | 5.35 | 0.96 (0.87, 1.06) | 491.70 (443.43, 545.23) | 0.96 |
|  | 6.79 | 1.18 (1.12, 1.24) | 642.98 (624.58, 661.93) | 0.99 |
|  | 8.09 | 1.66 (1.48, 1.89) | 226.36 (200.24, 255.89) | 0.92 |
|  | 9.72 | 0.94 (0.85, 1.05) | 6.11 (4.77, 7.83) | 0.95 |
| γ-CDP | 4.96 | 1.12 (1.08, 1.18) | 76.90 (71.97, 82.15) | 0.99 |
|  | 6.00 | 0.79 (0.77, 0.81) | 119.90 (115.51, 124.45) | 1.00 |
|  | 8.98 | 0.90 (0.86, 0.94) | 28.61 (26.26, 31.17) | 0.99 |
|  | 9.30 | 0.87 (0.84, 0.90) | 19.27 (17.72, 20.95) | 0.99 |
|  | 10.11 | 0.69 (0.67, 0.72) | 2.75 (2.44, 3.11) | 0.99 |
| β-HP-CDP | 5.76 | 0.59 (0.55, 0.64) | 552.33 (516.27, 590.91) | 0.97 |
|  | 6.57 | 1.02 (0.97, 1.08) | 737.40 (697.94, 779.08) | 0.99 |
|  | 6.88 | 1.21 (1.14, 1.30) | 364.42 (345.51, 384.36) | 0.98 |
|  | 8.13 | 1.60 (1.52, 1.70) | 239.11 (227.92, 250.85) | 0.98 |
|  | 9.51 | 1.05 (1.00, 1.11) | 8.23 (7.31, 9.25) | 0.99 |
| β-γ-CDP | 5.06 | 1.05 (0.98, 1.13) | 75.16 (67.02, 84.30) | 0.97 |
|  | 6.40 | 0.87 (0.85, 0.90) | 194.76 (188.09, 201.67) | 1.00 |
|  | 7.57 | 1.15 (1.10, 1.21) | 179.47 (171.07, 188.29) | 0.99 |
|  | 8.63 | 1.72 (1.56, 1.91) | 174.02 (155.87, 194.29) | 0.95 |
|  | 9.81 | 1.12 (1.08, 1.16) | 11.08 (10.27, 11.96) | 0.99 |
| γ-HP-CDP | 5.80 | 0.89 (0.84, 0.95) | 951.48 (891.03, 1016.04) | 0.98 |
|  | 6.46 | 1.04 (1.01, 1.07) | 720.61 (700.28, 741.53) | 1.00 |
|  | 7.24 | 0.97 (0.89, 1.06) | 880.85 (803.03, 966.21) | 0.97 |
|  | 9.30 | 2.04 (1.83, 2.32) | 75.44 (67.20, 84.69) | 0.93 |
|  | 10.07 | 1.09 (1.04, 1.14) | 7.27 (6.53, 8.09) | 0.99 |
