# Supplementary material for: Molecular Insights into the pH-Dependent Adsorption and Removal of Ionizable Antibiotic Oxytetracycline by Adsorbent Cyclodextrin Polymers
Source: PLoS One. 2014 Jan 21;9(1):e86228. doi: 10.1371/journal.pone.0086228 (PMC3897700; doi:10.1371/journal.pone.0086228)
Supplement: Table S10 — Linear correlation between K d values calculated and experimental. (DOC) [file pone.0086228.s014.doc]

**Table S10.** Linear correlation between *K*_d_ values calculated and experimental.

|  | Equation | *R*^2^ | P |
| --- | --- | --- | --- |
| β-CDP | *y*^a^=1.00*x*^b^ | 1.00 | < 0.01 |
| RMCDP | *y*=0.98*x* | 0.98 | < 0.01 |
| HPCDP | *y*=0.97*x* | 0.95 | < 0.01 |
| γ-CDP | *y*=0.88*x* | 0.86 | < 0.01 |
| β-HP-CDP | *y*=0.93*x* | 0.92 | < 0.01 |
| β-γ-CDP | *y*=0.89*x* | 0.86 | < 0.01 |
| γ-HP-CDP | *y*=0.95*x* | 0.96 | < 0.01 |

^a^ The values were calculated on the basis of Equation 2 into which the *K*_d_ and fraction of the species were substituted.

^b^ The values were obtained from batch adsorption experiments.
