# Supplementary figures and images for: Molecular Insights into the pH-Dependent Adsorption and Removal of Ionizable Antibiotic Oxytetracycline by Adsorbent Cyclodextrin Polymers
Source: PLoS One. 2014 Jan 21;9(1):e86228. doi: 10.1371/journal.pone.0086228 (PMC3897700; doi:10.1371/journal.pone.0086228)

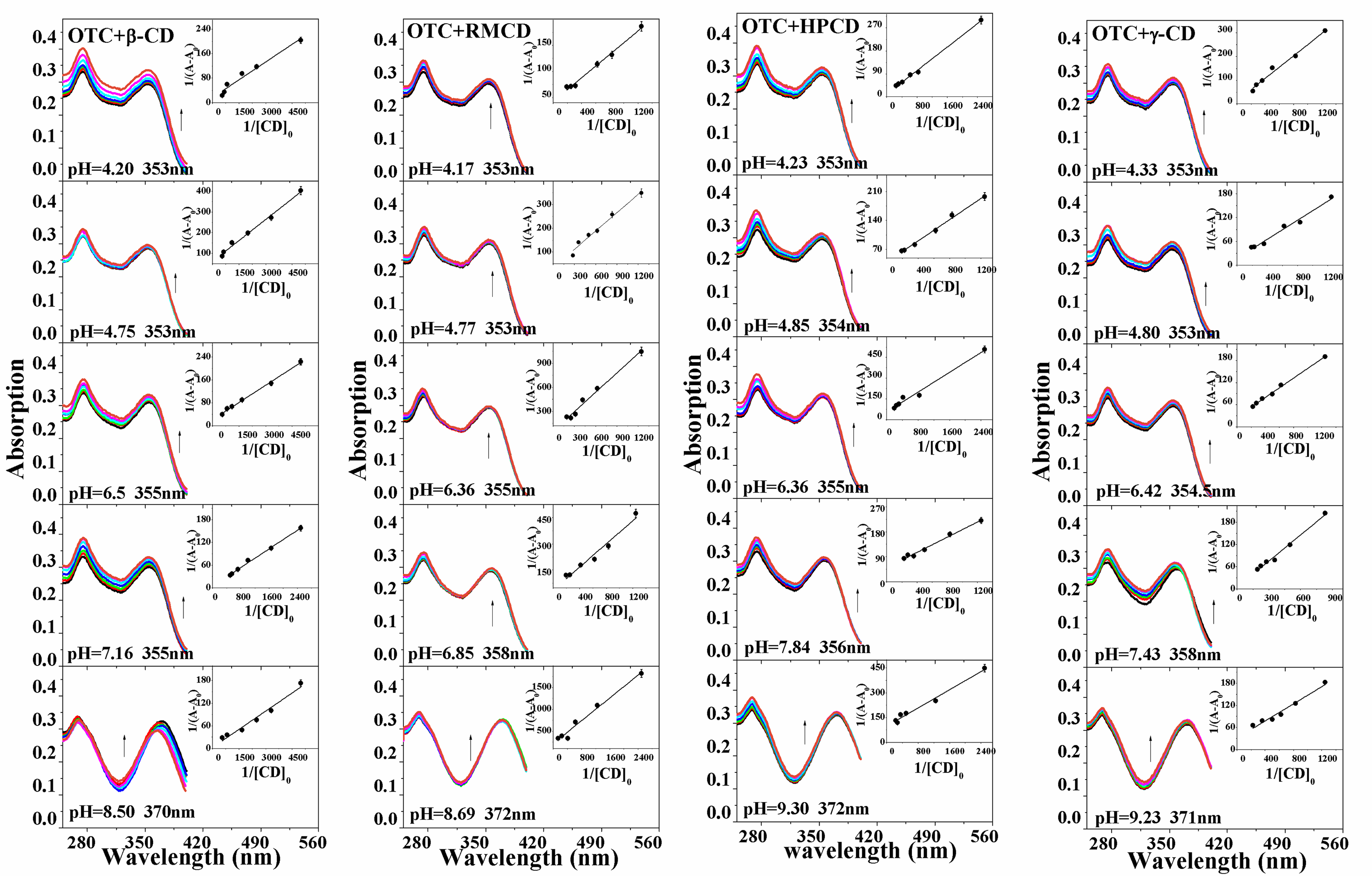

Supplement: Figure S1 — UV-Vis spectra of CD-OTC complexes at varying pH. (TIF) [file pone.0086228.s015.tif]

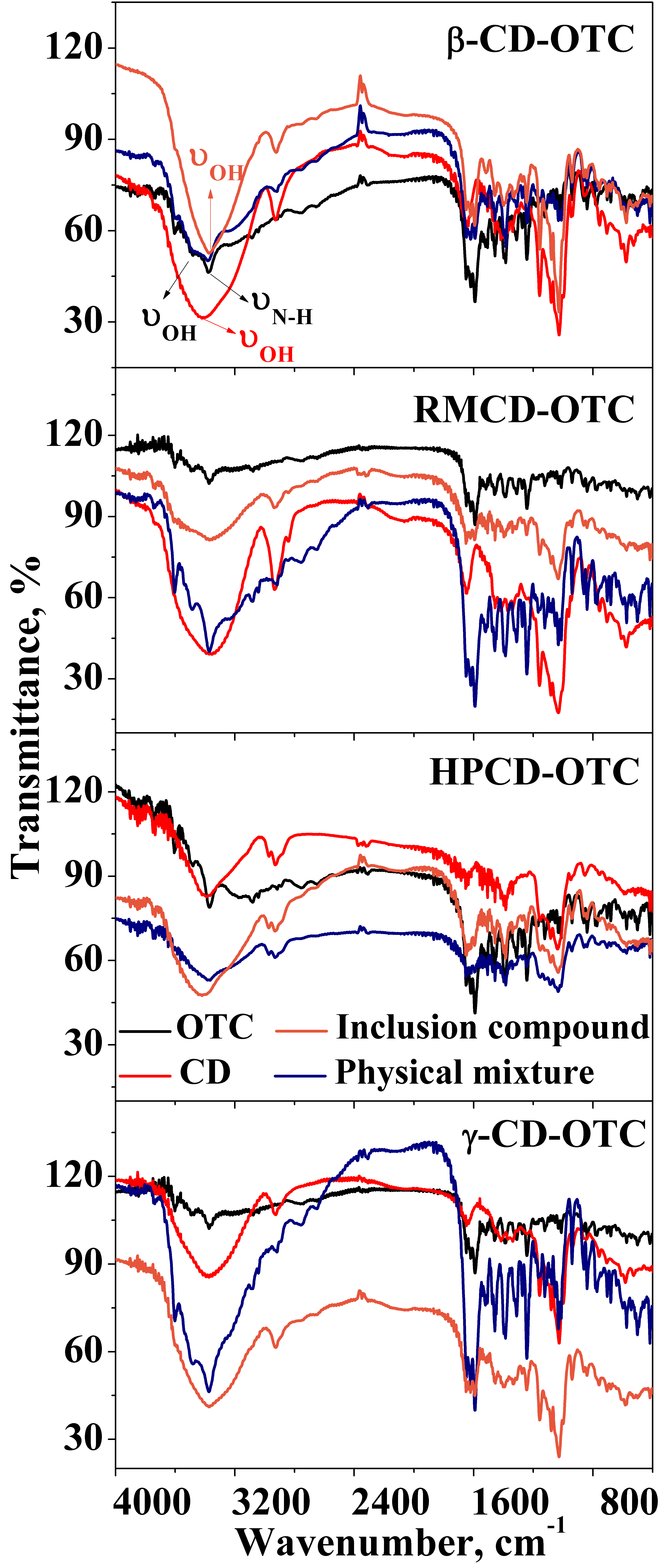

Supplement: Figure S2 — FTIR spectra of CD, OTC and CD-OTC complexes. (TIF) [file pone.0086228.s016.tif]

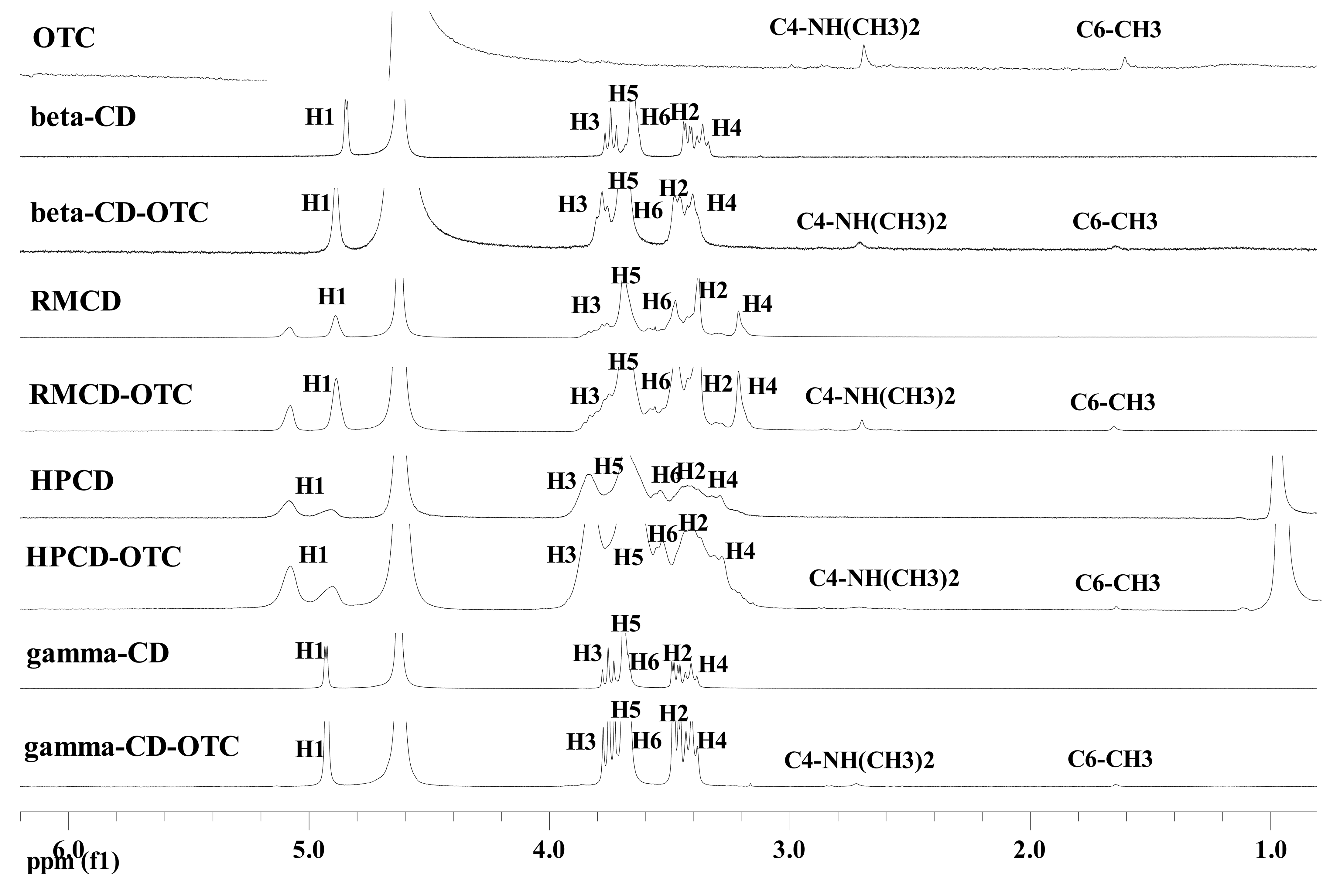

Supplement: Figure S3 — 1H NMR spectra of OTC, CD and CD-OTC complexes (dissolved in D2O). HOD at 4.617 ppm was taken as a reference. (TIF) [file pone.0086228.s017.tif]

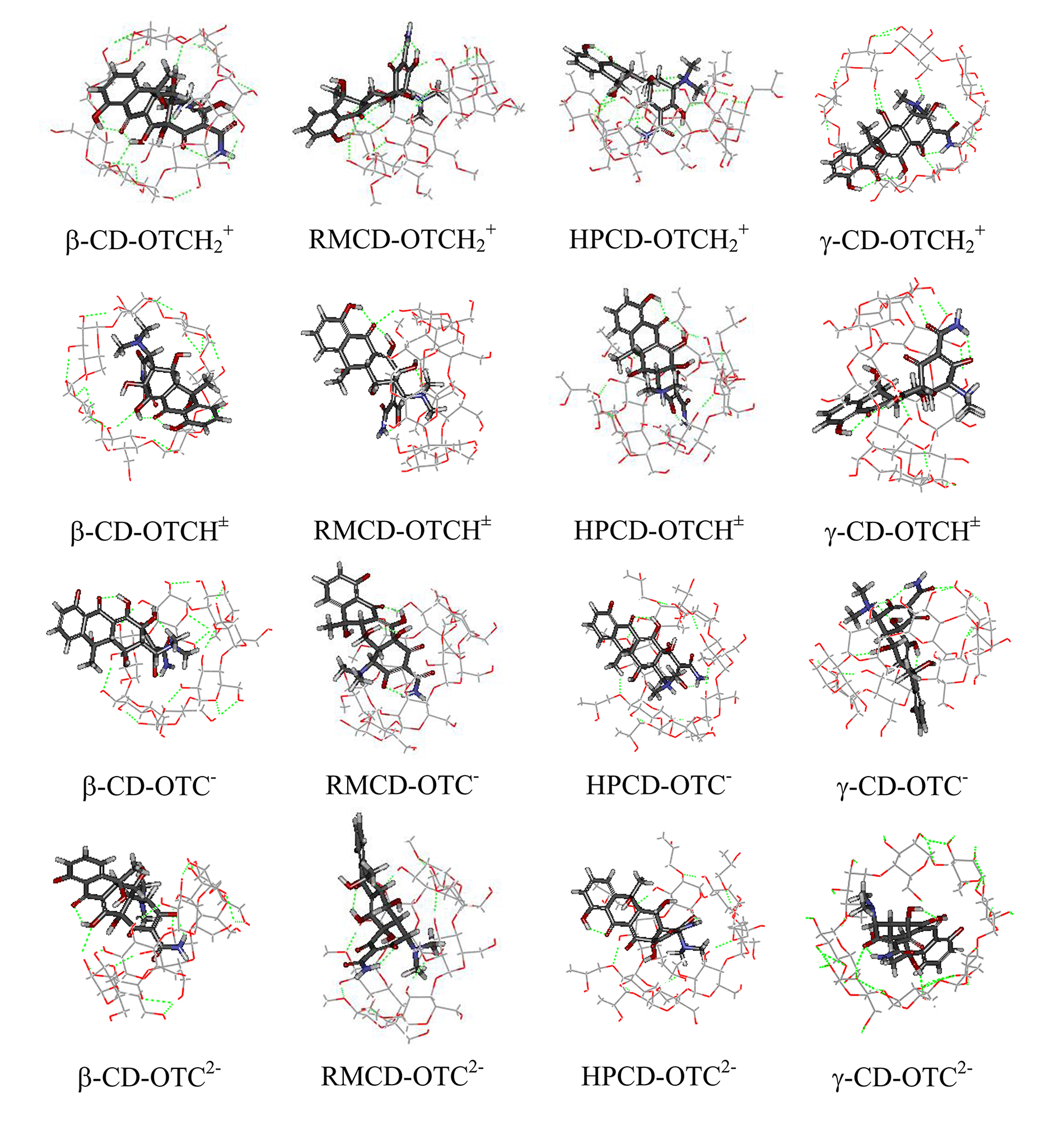

Supplement: Figure S4 — Molecular docking of CD with the species. The green dotted line represents hydrogen bonding. (TIF) [file pone.0086228.s018.tif]

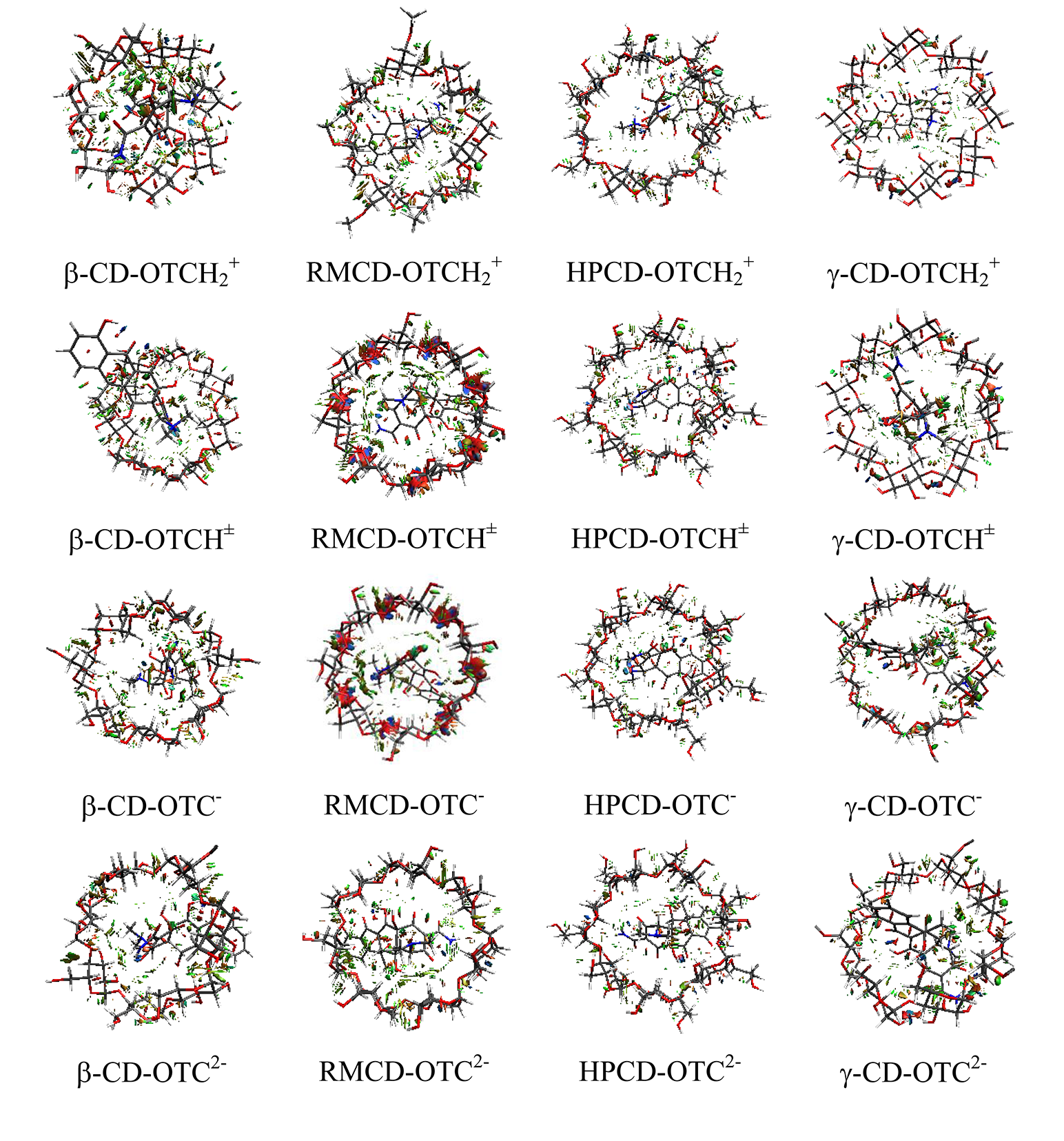

Supplement: Figure S5 — Qualitative comparison of intermolecular interactions between CD and the species. The blue, green, and red isosurfaces are indicative of hydrogen bonding, van der Waals forces, and repulsion interactions, respectively. (TIF) [file pone.0086228.s019.tif]

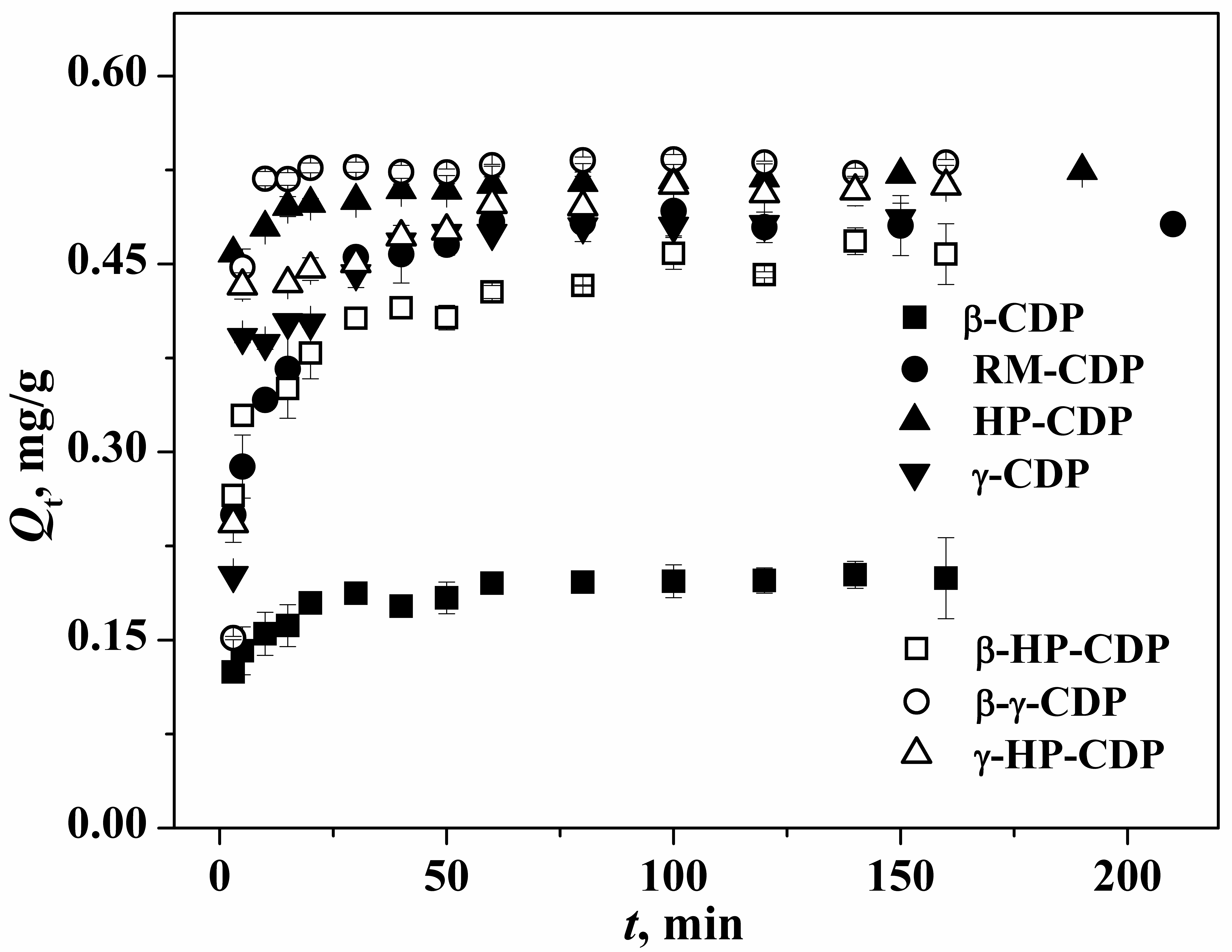

Supplement: Figure S6 — Adsorption kinetics of OTC onto CDPs at pH 7.0. (TIF) [file pone.0086228.s020.tif]

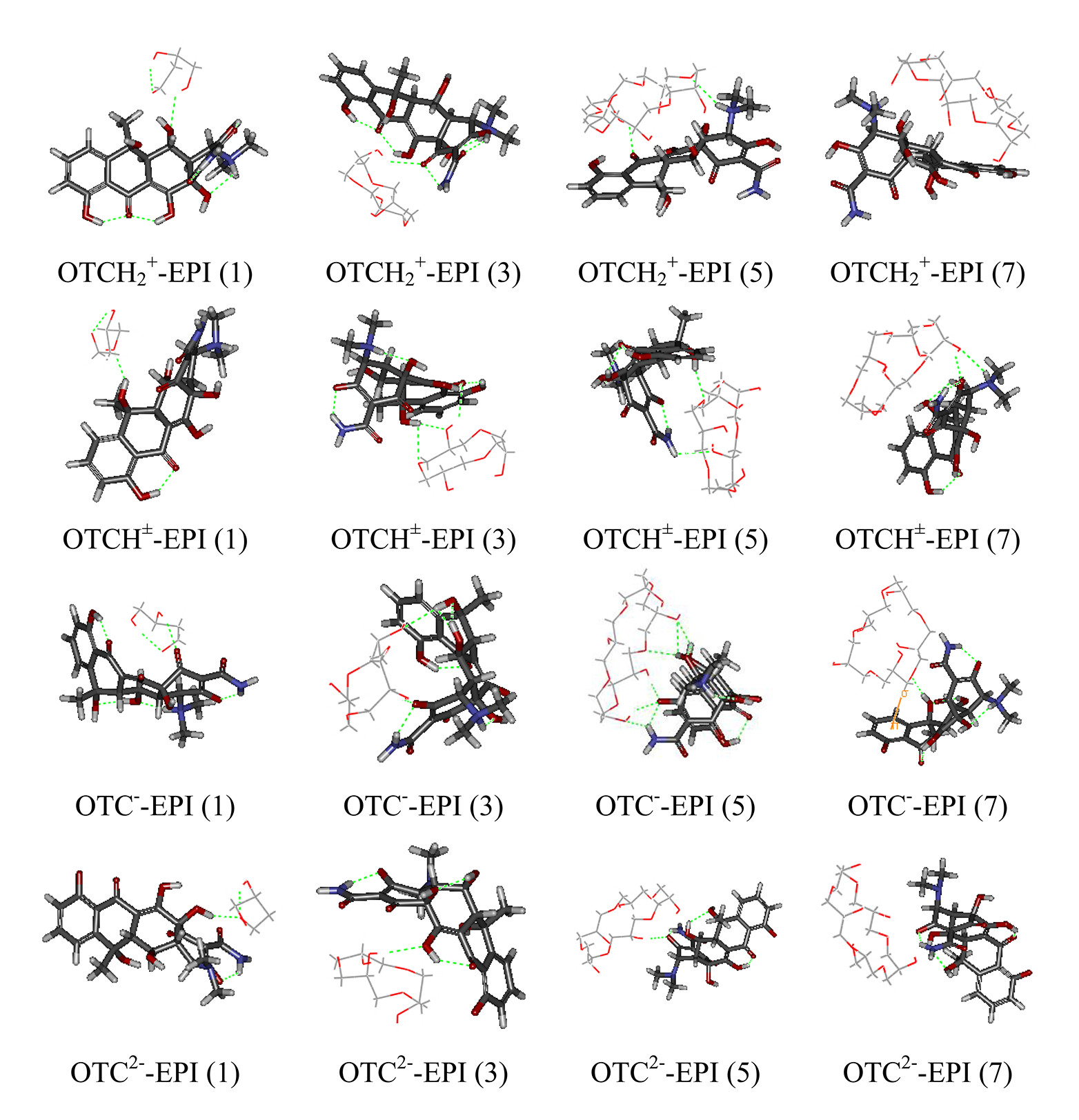

Supplement: Figure S10 — Molecular docking of the EPI oligomer with the species. The green dotted line represents hydrogen bonding and the orange line represents σ-π interaction. The numbers in parentheses represent ring-opened, tricyclo-, pentacyclo-, and heptacyclo-forms, respectively. (TIF) [file pone.0086228.s024.tif]
